# Phylogeography and population structure of the tsetse fly *Glossina pallidipes* in Kenya and the Serengeti ecosystem

**DOI:** 10.1371/journal.pntd.0007855

**Published:** 2020-02-24

**Authors:** Rosemary Bateta, Norah P. Saarman, Winnie A. Okeyo, Kirstin Dion, Thomas Johnson, Paul O. Mireji, Sylvance Okoth, Imna Malele, Grace Murilla, Serap Aksoy, Adalgisa Caccone

**Affiliations:** 1 Biotechnology Research Institute, Kenya Agricultural and Livestock Research Organization, Kikuyu, Nairobi, Kenya; 2 Department of Ecology and Evolutionary Biology, Yale University, Connecticut, United States of America; 3 Department of Biomedical Sciences and Technology, School of Public Health and Community Development, Maseno University, Maseno, Kisumu, Kenya; 4 Centre for Geographic Medicine Research Coast, Kenya Medical Research Institute, Kilifi, Kenya; 5 Vector and Vector Borne Diseases Research Institute, Tanzania Veterinary Laboratory Agency, Tanga, Tanzania; 6 Department of Epidemiology of Microbial Diseases, Yale School of Public Health, Connecticut, United States of America; International Centre of Insect Physiology and Ecology, KENYA

## Abstract

*Glossina pallidipes* is the main vector of animal African trypanosomiasis and a potential vector of human African trypanosomiasis in eastern Africa where it poses a large economic burden and public health threat. Vector control efforts have succeeded in reducing infection rates, but recent resurgence in tsetse fly population density raises concerns that vector control programs require improved strategic planning over larger geographic and temporal scales. Detailed knowledge of population structure and dispersal patterns can provide the required information to improve planning. To this end, we investigated the phylogeography and population structure of *G*. *pallidipes* over a large spatial scale in Kenya and northern Tanzania using 11 microsatellite loci genotyped in 600 individuals. Our results indicate distinct genetic clusters east and west of the Great Rift Valley, and less distinct clustering of the northwest separate from the southwest (Serengeti ecosystem). Estimates of genetic differentiation and first-generation migration indicated high genetic connectivity within genetic clusters even across large geographic distances of more than 300 km in the east, but only occasional migration among clusters. Patterns of connectivity suggest isolation by distance across genetic breaks but not within genetic clusters, and imply a major role for river basins in facilitating gene flow in *G*. *pallidipes*. Effective population size (N_e_) estimates and results from Approximate Bayesian Computation further support that there has been recent *G*. *pallidipes* population size fluctuations in the Serengeti ecosystem and the northwest during the last century, but also suggest that the full extent of differences in genetic diversity and population dynamics between the east and the west was established over evolutionary time periods (tentatively on the order of millions of years). Findings provide further support that the Serengeti ecosystem and northwestern Kenya represent independent tsetse populations. Additionally, we present evidence that three previously recognized populations (the Mbeere-Meru, Central Kenya and Coastal “fly belts”) act as a single population and should be considered as a single unit in vector control.

## Author summary

Tsetse flies are responsible for transmission of trypanosomes that cause human African trypanosomiasis (HAT) and animal African Trypanosomiasis (AAT) in sub-Saharan Africa, and are thus of economic importance in the regions they inhabit. In Kenya and Tanzania, control of the main vector species, *Glossina pallidipes*, plays an important role in control of both HAT and AAT. Understanding the population structure of *G*. *pallidipes* is important in designing effective fly control strategies. In this study, we determined the spatial genetic structuring of *G*. *pallidipes* on a broad spatial scale with genotype data from 11 microsatellite loci and 21 sampling sites in Kenya and northern Tanzania. Results indicate a strong regional separation of tsetse fly populations into three major genetic clusters, with divergence east and west of the Great Rift Valley in central Kenya and between the Serengeti ecosystem and other western sites. Findings from ABC simulations suggest that the east/west divergence reflects the biogeographic break across the Great Rift Valley, and the northwest/southwest divergence reflects the biogeographic break between low elevation savannah and the Kenyan highlands, both biogeographic breaks previously observed in savannah animals with similar ranges. We found evidence of high genetic connectivity and migration rates within each of the three genetic clusters, and only occasional migration between clusters. These patterns of genetic connectivity and migration suggest that, following local eradication, the risk of reinvasion from within genetic clusters is very high even across vast geographic distances of more than 300 km, and that the risk of reinvasion from different genetic clusters is much lower, but still of concern. We argue that these findings call for tsetse control strategies that are coordinated for each genetic cluster, and monitoring schemes that are specifically designed to detect migration events across the geographic boundaries that demarcate genetic clusters. Although coordination of control within and monitoring between genetic clusters will be challenging because of the large spatial extent of the east genetic cluster and the international scale of the Serengeti ecosystem, we argue it is necessary to prevent reinvasions from both proximal and distant localities.

## Introduction

Tsetse flies (genus *Glossina*) are restricted to Sub-Saharan Africa and inhabit patchy and discontinuous habitat within their distribution [1,2]. In Kenya and Tanzania [3,4], *Glossina pallidipes* is the most widely-distributed vector of trypanosomes that cause Animal African Trypanosomiasis (AAT), and to a lesser degree, has also been involved in Human African Trypanosomiasis (HAT) transmission [5,6]. Distribution of G. *pallidipes* runs from Ethiopia to Kenya, Uganda, Tanzania, Democratic Republic of the Congo, Mozambique and Zambia, [7–9] and its population density depends on the availability of a suitable habitat and mammalian hosts [10]. The presence, distribution, and abundance of tsetse flies depends on availability of an appropriate habitat [11]. The extent of the spatial distribution of tsetse matches changes in seasons, where tsetse populations reduce in size during several arid periods of the year, but increase in size during rainy seasons [12–14].

**Fig 1:**
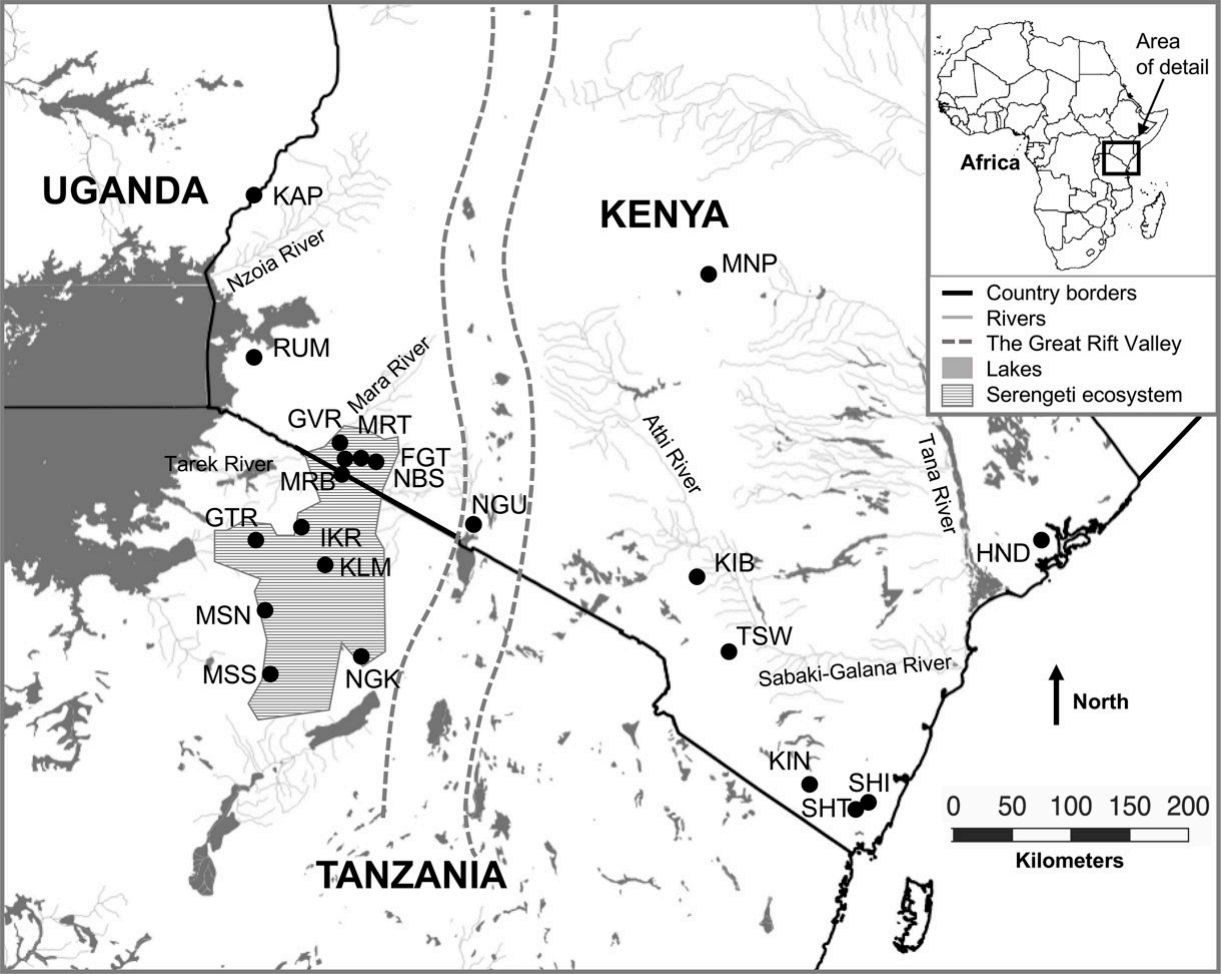
Map showing sample sites in Kenya and Tanzania and location of the study region within Africa. Sampling sites shown with dots and labeled with three letter codes as listed in Table 1. The striped region denotes the 11 sampling sites within the Serengeti ecosystem. The map was created in QGIS v2.12.1 (August 2017; http://qgis.osgeo.org) with free and publicly available data from DIVA-GIS (August 2017; http://www.diva-gis.org).

Geographical Information System (GIS) prediction models have been used to show areas of tsetse abundance and expansion [13,15], and these models suggest that several human and natural disturbance have impacted tsetse distribution at various times. This finding is supported by population genetic analyses that indicated genetic shifts [16] especially in regions where human activities have altered conditions [17,18]. Our previous study showed that tsetse control efforts during the 1960s and 1980s (African union 2009) [19] did not interfere with the genetic diversity of tsetse [16]. Over longer time intervals, disease epidemics such as the Rinderpest outbreak that occurred in the early 1990s [20,21], and the biogeographic break caused by the formation of the Ethiopian rift (the section of the Great Rift Valley in central Kenya; Fig 1), were also likely to have impacted the genetic differentiation of tsetse flies, as it did for many other groups of animals and plants [22–25]. Understanding the relative impact of these various biogeographic forces is important for the development and coordination of effective and feasible vector control strategies in Kenya and Tanzania, two countries that are heavily burdened by the economic cost of AAT.

The goal of this study was to evaluate patterns and levels of genetic connectivity of *G*. *pallidipes* across multiple spatial scales, and to understand the evolutionary forces that have shaped and maintained them. We used samples collected from 21 sites in Kenya and the Serengeti National Park in Tanzania (Fig 1) and screened approximately 600 samples for genetic variation at 11 microsatellite loci. Sampling covered five of the eight tsetse fly belts recognized by the Kenya Tsetse and Trypanosomiasis Eradication Council (KENTTEC). Using KENTTEC recommended terminology, we included samples from the Lake Victoria basin fly belt (KAR and RUM), the Narok-Kajiado fly belt (Maasai Mara National Reserve within the Serengeti ecosystem: GVR, MRT, FGT, NBS and MRB), the Mbeere-Meru fly belt (MNP), the Central Kenya fly belt (KIB), and the Coastal fly belt (HND, TSW, KIN, SHI and SHT). We investigated the pattern of genetic structure over three spatial scales, with higher resolution than previous genetic analyses of *G*. *pallidipes* [26–30]. At the largest spatial scale (Fig 1), we investigated genetic structure from 21 sites across the *G*. *pallidipes* distribution in Kenya and the Serengeti National Park in Tanzania. At the intermediate spatial scale, we investigate 13 and eight sites that fall west and east (respectively) of the Great Rift Valley ‘Ethiopian rift’, which is a biogeographic boundary that marks a genetic break in *G*. *pallidipes* [26]. At the smallest spatial scale, we investigated 11 sites within the Serengeti ecosystem, which is one of the largest expanses (~25,000 km^2^) of well-connected natural savannah habitat in the world [31]. The Serengeti ecosystem is the iconic site of one of the best-known periodical migrations of large vertebrates that spans the Kenya/Tanzania border [32]. The ecosystem is protected in both Kenya and Tanzania, by the Maasai Mara National Reserve and the Serengeti National Park, respectively. Findings from our investigation at three spatial scales in Kenya and Tanzania can help develop effective vector control and monitoring strategies to coordinate efforts at local, regional, national, and international spatial scales.

## Methods

### Ethics Statement

Field collections of tsetse flies were conducted under permit number NACOSTI/P/18/28381/22226 granted by the Kenya National Commission for Science, Technology and Innovation.

### Study sites and tsetse samples

Biconical [33] and Ngu [34] traps were used to collect tsetse flies from twenty-one sampling sites during the time period of March 2015 through November 2016 (Fig 1, Table 1). To ensure trapping effort was uniform among sites, in each location 7–15 traps were placed within a 1 km radius at least 150 m apart from one another and emptied after 24 hrs. Flies were preserved individually in 1.5 mL tubes containing 80% ethanol. The collection date, trap number and coordinates, and sex were recorded on each sample tube. *Glossina pallidipes* samples were collected from 21 sites across Kenya and northern Tanzania (Fig 1), including from 11 sites within the Serengeti ecosystem and eight sites from a previous study [26]. A total of 600 tsetse flies were genotyped, representing ~30 flies per site except for two locations (SHI and SHT), which had 22 and 8 flies, respectively. To avoid possible sex-bias, the same number of males and females were included.

**Table 1:** Sampling sites and estimates of their genetic diversity and assignment.

Site (date)	Code	Lat.	Long.	N	AR	H_O_	H_E_	F_IS_	F_IS_ p-value	q^NW^	q^SW^	q^E^	q^O^
**Northwest**													
Kapesur (Jun 2016)	KAP	0.733	34.316	30	2.06	0.42	0.51	0.18	0.00	0.93	0.00	0.00	0.07
Ruma (Jan 2016)	RUM	-0.608	34.307	30	1.81	0.38	0.42	0.10	0.02	1.00	0.00	0.00	0.00
**Southwest (*Serengeti Ecosystem*)**													
Governor’s Camp (Aug 2016)	GVR	-1.309	35.034	30	2.3	0.49	0.58	0.16	0.00	0.00	1.00	0.00	0.00
Mara Talek (Aug 2015)	MRT	-1.431	35.059	30	2.26	0.54	0.56	0.04	0.09	0.03	0.97	0.00	0.00
Fig Tree Camp (Aug 2016))	FGT	-1.436	35.194	30	2.36	0.54	0.59	0.09	0.01	0.09	0.89	0.02	0.00
Naibosho (Sept 2016)	NBS	-1.465	35.309	30	2.29	0.51	0.57	0.10	0.00	0.00	1.00	0.00	0.00
Marabridge (Aug 2016)	MRB	-1.556	35.025	30	2.44	0.52	0.62	0.16	0.01	0.00	1.00	0.00	0.00
Grumeti (Nov 2016)	GTR	-2.092	34.322	30	2.39	0.55	0.61	0.10	0.00	0.00	1.00	0.00	0.00
Ikorongo (Nov 2016)	IKR	-2.026	34.692	30	2.42	0.54	0.62	0.13	0.00	0.00	0.93	0.00	0.07
Kilimafedha (Oct 2016)	KLM	-2.299	34.901	30	2.43	0.58	0.63	0.08	0.00	0.00	0.98	0.02	0.00
Maswa North (Nov 2016)	MSN	-2.674	34.401	30	2.46	0.55	0.63	0.13	0.00	0.00	1.00	0.00	0.00
Maswa South (Nov 2016)	MSS	-3.199	34.46	30	2.43	0.58	0.62	0.07	0.03	0.01	0.98	0.02	0.00
Ngorongoro (Oct 2016)	NGK	-3.446	34.886	30	2.35	0.54	0.59	0.08	0.01	0.00	0.97	0.03	0.00
**East**	** **	** **	** **	** **	** **	** **	** **	** **	** **	** **	** **	** **	
Nguruman (Mar 2015)	NGU	-1.977	36.117	30	2.12	0.51	0.54	0.06	0.04	0.03	0.10	0.87	0.00
Meru National Park (Jan 2016)	MNP	0.077	38.064	30	2.5	0.58	0.65	0.11	0.00	0.00	0.00	1.00	0.00
Kibwezi (Oct 2016)	KIB	-2.416	37.954	30	2.53	0.58	0.66	0.12	0.00	0.00	0.00	1.00	0.00
Tsavo west (Aug 2015)	TSW	-3.027	38.218	30	2.55	0.57	0.65	0.13	0.00	0.00	0.00	1.00	0.00
Kinango (Aug 2015)	KIN	-4.108	38.874	30	2.55	0.55	0.65	0.16	0.00	0.00	0.02	0.98	0.00
Tiribe (Aug 2015)	SHT	-4.338	39.264	8	2.51	0.4	0.72	0.48	0.00	0.00	0.00	1.00	0.00
Shimba (Aug 2015)	SHI	-4.152	39.420	22	2.54	0.52	0.66	0.22	0.00	0.00	0.00	1.00	0.00
Hindi (Jun 2016)	HND	-2.117	40.791	30	2.54	0.56	0.65	0.14	0.00	0.00	0.03	0.97	0.00

Sampling information including sampling site (and date of collection), code, latitude (Lat.), longitude (Long.), number of samples (N), mean allelic richness across all 11 loci (AR), observed heterozygosity (H_O_), expected heterozygosity (H_E_), inbreeding coefficient (F_IS_), and results from the STRUCTURE [35,36] clustering analysis of average assignment probability (q) to the northwest, southwest, east, and outlier clusters (q^NW^, q^SW^, q^E^, q^O^, respectively).

To evaluate the genetic structure of these populations at a country-wide scale we included samples from across the species current distribution in Kenya (Fig 1; Table 1). We also visited 14 more locations that did not have any flies, despite past collection records that indicated the presence of *G*. *pallidipes* (S1 Table). Absence of flies in these 14 localities could have been caused by recent land use changes that have altered the habitat for agricultural use. To investigate patterns of genetic structure within the Serengeti ecosystem we used samples from five sites (GVR, MRT, FGT, NBS and MRB) from the Maasai Mara National Reserve in Kenya and six sites (GTR, IKR, KLM, MSN, MSS, and NGK) from the Serengeti National Park in Tanzania.

### DNA extraction, microsatellite genotyping and mtDNA sequencing

DNA was extracted from two legs per fly using either the PrepGEM insect DNA (ZYGEM Corp Ltd, Hamilton, New Zealand) or the Qiagen DNAeasy blood and tissue (Qiagen, Hilden, Germany) extraction kits, following manufacturers’ protocol. We used fluorescent labelled (FAM, TAM, HEX and NED) forward primers for 11 microsatellite loci (S2 Table) using published protocols that had been validated for *G*. *pallidipes* [16]. Briefly, PCR amplifications were carried out in a Mastercycler Pro Thermocycler (Eppendorf, Germany) in 13 μL reactions consisting of 6 μl of distilled H_2_0, 1.1μl of 25 mM MgCl_2_, 0.5 μL each of 10mM forward and reverse primers, 0.1 μl of 100X BSA, 1.1 μl of 10 mM dNTP mix, 1μl of DNA template and 0.1 μl of 5 U/μl GoTaq DNA polymerase with 2.6 μL of 5X PCR Buffer (Promega, USA). We used the following cycling conditions: 95°C for five minutes, 12 touch-down cycles (95°C for 30 seconds, 60–50°C for 25 seconds, and 72°C for 30 seconds), 40 additional cycles (95°C for 30 seconds, 50°C for 25 seconds, and 72°C for 30 seconds), and a final extension of 72°C for 20 minutes. PCR products were multiplexed in groups of two or three loci in the same way as previously published [16], and genotyped on an ABI 3730xL Automated Sequencer (Life Technologies, USA) at the DNA Analysis Facility on Science Hill at Yale University (http://dna-analysis.yale.edu/). Alleles were scored using the program GENEMARKER v2.4.0 (Soft Genetics, USA). To ensure replication of genotype calls, automatically generated peaks were visually inspected twice independently using agreed upon criteria for each locus (S1 File), and only genotype calls that agreed were retained.

For approximate Bayesian computation analysis exploring potential causes of population structure, we sequenced a 439 bp fragment of mitochondrial DNA (mtDNA) from the cytochrome oxidase I gene was PCR-amplified in 24 individuals using primers designed by Simon *et al* [37] (sequencing details are in S2 File). Geneious v6.0.6 software [38] was used to edit and align sequences, and unique mtDNA haplotypes and evolutionary relationships between haplotypes were constructed with parsimony-based network using TCS var 1.21 software [39] as implemented in PopART (Population Analysis with Reticulate Trees: http://popart.otago.ac.nz/index.shtm).

### Microsatellite marker validation and diversity

We checked for presence of null alleles using Micro-Checker v2.2.3 [40] and loci with evidence of null alleles in all sampled sites were dropped from subsequent analyses. We tested all microsatellite loci for linkage disequilibrium and deviation from Hardy-Weinberg equilibrium using Genepop v4.6 [41]. All loci were evaluated using the Markov chain method [42] with 10,000 dememorization steps, 1000 batches, and 10,000 iterations per batch. Fisher’s method was used to obtain significance values that were adjusted for multiple tests using the Benjamini-Hochberg method ([43]). We used Arlequin *v3*.*5*.*2*.*2* [44] to determine observed (Ho) and expected heterozygosity (He). Allelic richness (AR) and inbreeding coefficient (F_IS_) were calculated using FSTAT v2.9.3.2 [45].

### Estimates of effective population size (N_e_) and population bottlenecks

We assessed population dynamics with estimates of effective population size (N_e_), which can be considered a proxy for the amount of variation present in the population, and tests for recent population bottlenecks. These parameters inform the numbers of breeding individuals in a region, the effective dispersal ability, the potential strength of selection for resistance to vector control manipulations (genetic or para-genetic engineering or release of sterile males) [46–49]. Thus, improved understanding of these parameters can help to model transmission dynamics and inform on-the-ground vector control strategies. We used one-sample linkage disequilibrium method [50], implemented in NeESTIMATOR v2 [51]. We tested for recent bottlenecks in BOTTLENECK 1.2.02 [52], a program that can detect bottlenecks approximately 2N_e_ to 4N_e_ generations before sampling [52,53]. We tested for excess heterozygosity compared to observed allelic diversity using the Wilcoxon’s one tailed signed rank test [52] under the two-phase mutation model (TPM) with 70% single-step mutations and 30% multiple-step mutations, and the infinite allele model (IAM), both with 10,000 iterations. We reported the raw p-values, and p-values that were adjusted for multiple tests using the Benjamini-Hochberg method ([43]). The TPM and IAM models differ in their degree of mutation approximation, with the TPM model generally considered the most appropriate for microsatellite data [54]. We also included the IAM model for comparison but a population was considered having undergone a recent bottleneck only if there was a consensus by both models. The mode shift function of BOTTLENECK was employed to determine allele frequency distributions and infer whether distortions in distributions were likely to be bottleneck-induced [53].

### Population structure

We used the Bayesian clustering method implemented in BAPS v 6 [55,56] to investigate the overall population structure among all sampling sites while accounting for geographic origin of each sample with the “spatial clustering of individuals” option. This method outperforms clustering methods when sampling is uneven across the landscape and/or there is isolation by distance [57–60]. We ran 10 independent replicates of the initial clustering step with a maximum number of clusters (K) of 21 (the number of sampling sites), 10, and 5 to ensure stability of results as recommended by the authors of the method [55,56]. We then estimated admixture that reflect the probability of each individual belonging to distinct genetic units (q-values ranging from 0 to 1) in all individuals clustering using 50 reference individuals from each cluster identified in the “spatial clustering of individuals” analysis using 10,000 iterations. For comparison of BAPS results with a second Bayesian method that did not account for geographic origin, we also ran STRUCTURE *v2*.*3*.*4* [35,36] with K = 1–10, the admixture model, independent allele frequencies, and a burn-in of 50,000 followed by 250,000 steps, and used CLUMPAK [61] to align the 10 independent replicates for each K.

To further visualize patterns of genetic structure, we also performed principal components analysis (PCA) and discriminant analysis of principal components (DAPC) [62] in the "adagenet" package v2.1.0 [63] in R v3.3.3 (R Development Core Team), which are both model-free multivariate procedures that (unlike BAPS and STRUCTURE) make no assumptions about compliance with Hardy Weinberg equilibrium.

We measured genetic divergence among sampling localities by computing pairwise F_ST_ using Arlequin with Wright’s statistics [64] and tested for significance using the variance method developed by Weir and Cockerham [65], computed at 10,000 permutations to obtain exact p-values. The resulting F_ST_ values and geographic distances generated by using the web based Geographic Distance Matrix Generator v1.2.3 (Ersts, http://biodiversityinformatics.amnh.org/open_source/gdmg, Internet) were used to test for isolation by distance with following Rousset 1997 [66] using F_ST_/(1- F_ST_) and log transformed geographic distance in Km as implemented in the “isolation by distance” web service v3.23 [67] with a Mantel test with 10,000 randomizations [68].

### Relatedness and migration

Relatedness between individuals within genetic clusters was tested using the program ML-Relate [69] to determine whether the observed genetic clustering was a result of sampling related individuals. We assigned pairwise relationships within each population into one of four relationship categories; unrelated (U), half siblings (HS), full siblings (FS) or parent/offspring (PO).

To test for individual migrants between geographically neighboring sampling sites, we used two methods. We used GENECLASS v2.0 [70–73] to detect first generation migrants. We used the Monte Carlo resampling algorithm of Paetkau *et al*., 2004 [71] with 1000 randomizations to compute the test statistics L_h_ (the likelihood of an individual’s assignment to the locality where it was sampled), L_max_ (the highest likelihood among all population sampled), and their ratio (L_h_/L_max_) to identify migrants. We used the Bayesian method of Rannala and Mountain, 1997 [72] to detect true migrants with a p-value cut-off of 0.05. We reported raw p-values and p-values that were adjusted for multiple tests using the Benjamini-Hochberg method ([43]).

### Biogeographic modeling with ABC

Population structure can have multiple causes including the slow accumulation of genetic differences across geographic space because of prolonged migration-drift equilibrium, or by genetic divergence across a geographic break (vicariance). Since the cause of structure remains unclear and has distinct implications on vector control, we explored the cause by modeling the timing of divergence of the major genetic clusters identified in BAPS with Approximate Bayesian Computation (ABC) in DIYABC [74] v2.0.4 ABC analysis was completed with two datasets: a subset of the existing microsatellite dataset, and a 439 bp mitochondrial DNA (mtDNA) fragment sequenced in 24 individuals for this purpose. We added the mtDNA dataset to allow inference of evolutionary history in the more distant past since mtDNA has slower mutation rates than microsatellites. DIYABC simulations assumed no migration between lineages and panmictic populations, so we used individuals from each major cluster that had no evidence of admixture or of being a migrant (northwest, southwest, east; see results section for full description of these clusters). In the microsatellite dataset, the three genetic clusters were represented by 50 individuals each (25 per site) from KAP and RUM (northwest), GTR and MSS (southwest), and MNP and KIB (east), respectively. In the mtDNA dataset, the three genetic clusters were represented by five individuals from RUM (northwest), 10 individuals from GTR (southwest), and nine individuals from KIB (east).

Priors for all parameters (S3 Table) allowed for a wide range of possibilities that were in line with estimates of mutation rates [75–79], population sizes [16,26,28,29,80–82], generation time [74, 82–84], and timing of population splits [25,83–86] made in previous studies of *G*. *pallidipes* and savannah species from east Africa (S2 File). We completed two analyses that made unique comparisons of alternative scenarios. Analysis 1 was designed to identify the most likely ancestral lineage and compared four scenarios (1a, 2a, 3a, and 4a; S1 Fig), while Analysis 2 was designed to distinguish between the likely timing of splits and N_e_ by comparing two scenarios with each of the possible patterns of ancestry (1a vs 1b, 2a vs 2b, 3a vs 3b, and 4a vs 4b; S1 Fig). We assessed the accuracy of scenarios by comparing summary statistics such as diversity, M-index [44,87], differentiation [53] (S2 File), and by then performing PCA with these statistics to estimate the relative posterior probability of alternative scenarios with the weighted logistic regression method described by [88]. We also estimated the posterior predictive error (frequency of accepting a scenario other than the true scenario) with 1000 runs model-checking using the method described by [74] to confirm reliability of the models, and made parameter estimates by drawing from the linear regression of the 1% of the simulations that were closest to the observed data.

## Results

### Microsatellite marker validation and mtDNA sequences generated for ABC analysis

*Glossina pallidipes* were genotyped at a total of 11 loci for 21 sampling sites for a total of 600 flies. We observed 49 instances of significant deviation from HWE after correcting for false discovery rate, using the Benjamin Hochberg method [43]. However, none of these loci showed a consistent pattern of deviations from HWE across all sampling sites (S4 Table), nor was there evidence of LD among loci (S5 Table). The 24 mtDNA sequences generated for the ABC analysis fell into 10 haplotypes, with the most common haplotype being present the three groups of samples chosen to represent the northwest, southwest, and east genetic clusters (see population structure results below for description of these clusters). All other nine haplotypes were unique to a single cluster (S2 Fig; S2 File).

### Genetic diversity and demographic estimates

Diversity statistics are shown in Table 1. Mean allelic richness across all loci was highest in both TSW and KIN (2.55) and lowest in RUM (1.81). RUM also presented the lowest H_o_ and H_e_ values, 0.38 and 0.42, respectively. H_o_ was highest in four sites (0.58: MSS, KLM, KIB, MNP), while the maximum H_e_ was observed in SHT (0.72). All sample sites revealed positive and significant F_IS_ values (p < 0.05). The lowest F_IS_ value (0.04) was observed in MRT while the highest (0.48) was observed in SHT. Estimates of Ho, H_e_, and F_IS_ indicate a small heterozygote deficit compared to what is expected under random mating.

Estimates of mean allelic richness after eliminating closely related individuals (see below) ranged from 1.87 in RUM to 2.62 in SHT (S6 Table) and reflected results obtained using the complete data set. RUM consistently presented the lowest H_o_ and H_e_ values (0.42 and 0.45, respectively), as observed using the complete data set. The highest H_o_ and H_e_ values 0.59 and 0.76 were observed in HND and SHT respectively. F_IS_ estimates for this subset data ranged from 0.06 in RUM to 0.45 in SHT (S6 Table), and remained significantly greater than zero except for RUM, indicating that individuals were more related on average than would be expected under a model of random mating, even after eliminating closely related individuals.

N_e_ estimates ranged widely from 2.7 (2.2–3.3 95% confidence interval [CI]) in KAP to 3,507 (125.8-infinite 95% confidence interval [CI]) in HND (Table 2). Some estimates were indistinguishable from infinity, indicating insufficient power to estimate N_e_ for these sampling sites.

**Table 2:** Estimates of effective population size (N_e_), bottleneck, and relatedness.

Site	N_e_	N_e_ 95% CI	p-value (TPM)	p-value (IAM)	AFD	% UR	% HS	% FS	% PO
KAP	2.7	2.2–3.3	0.58	0.06	L-shaped	70.6	13.6	4.8	11.0
RUM	n/a	82.6-∞	0.29	**0.03**	L-shaped	80.5	11.3	3.0	5.3
GVR	111.3	50 - ∞	0.90	0.29	L-shaped	85.7	11.7	1.6	0.9
MRT	626.1	81.8 - ∞	0.86	0.29	L-shaped	84.6	12.0	0.9	2.5
FGT	203.4	60.5 - ∞	0.45	0.09	L-shaped	85.1	12.2	1.6	1.1
NBS	n/a	279.7 - ∞	0.94	0.16	L-shaped	86.2	11.0	1.1	1.6
MRB	50.4	32.3–98.1	0.84	0.42	L-shaped	87.1	8.7	2.3	1.8
GTR	41.8	27.1–77.6	0.77	0.29	L-shaped	84.6	12.6	0.9	1.8
IKR	21.1	15.8–29.3	0.74	**0.03**	L-shaped	83.4	13.1	0.9	2.5
KLM	n/a	156.4 - ∞	0.45	**0.03**	L-shaped	86.4	11.7	0.9	0.9
MSN	21.3	16.1–29.4	0.71	0.12	L-shaped	84.8	12.4	1.4	1.4
MSS	94.8	43.6–22768.4	0.16	0.00*	L-shaped	85.5	12.4	1.6	0.5
NGK	111.9	48.6-∞	0.55	0.12	L-shaped	85.1	11.7	0.9	2.3
NGU	n/a	204.5-∞	0.84	0.10	L-shaped	79.0	14.0	2.7	4.3
MNP	86.6	646.1	0.23	**0.05**	L-shaped	84.6	13.8	1.1	0.5
KIB	49.2	33.3–85.4	0.82	0.14	L-shaped	89.9	9.0	0.7	0.5
TSW	n/a	444.8-∞	0.45	**0.03**	L-shaped	90.1	9.2	0.7	0.0
KIN	n/a	2252.5-∞	0.65	0.12	L-shaped	91.3	7.6	1.1	0.0
SHT	n/a	29.3 - ∞	0.54	0.14	L-shaped	100.0	0.0	0.0	0.0
SHI	n/a	209.4-∞	0.78	0.42	L-shaped	92.2	6.9	0.4	0.4
HND	3507.0	125.8 - ∞	0.82	0.10	L-shaped	90.0	8.3	1.2	0.5

Site, N_e_ estimates (marked n/a if indistinguishable from infinity), the N_e_ 95% confidence interval (CI), p-value of tests for bottlenecks under the TPM, and IAM mutation models, allele frequency distribution (AFD), and the percent of each sample that was estimated to be unrelated (UR), half-siblings (HS), full-siblings (FS), and part of a parent/offspring relationship (PS) is also reported. N_e_ was estimated with the LD method in NeESTIMATOR [51], tests for population bottlenecks were run in BOTTLENECK [52], and relatedness was estimated in ML-Relate [69]. Significant at p-value < 0.05 after Benjamini-Hochberg correction for multiple testing are marked *.

Results from the TPM model did not show a significant reduction in effective population size in any of the sample sites (Table 2), while the IAM model indicated a population bottlenecks in RUM, MSS, IKR, KLM, TSW and MNP (p < 0.05), but after correcting for multiple testing, only MSS was significant (Table 2). Similarly, there were no deviations from the normal L-shaped allele frequency distribution, indicating mutation-drift equilibrium and no population bottlenecks.

Maximum likelihood tests for relatedness indicate that the majority of individuals were unrelated (>70%; Table 2). The percentage of half sibling individuals ranged from 0% in SHT to 14% in NGU, with an overall average of 10%. Full sibling, ranging from 0% in SHT to 4.8% in KAP, with an overall average of 1.42%. Parent offspring ranged from 0 in SHT and 11% in KAP, with an overall average of 1.9%. These results indicate generally lower relatedness in the east than the west.

### Population structure and differentiation

**Fig 2:**
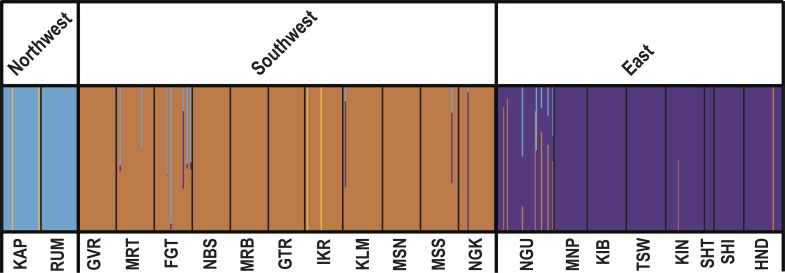
Results of the Bayesian clustering analyses based on microsatellite data. Spatially explicit genetic clustering was performed in the program BAPS v 6 [55,56] Vertical bars indicate the probability of assignment (q-value) of an individual to each cluster (S7 Table). Thin vertical lines separate sampling sites reported along the bottom x-axis, and think vertical lines separate the three major clusters reported along the top x-axis.

Bayesian analysis of population structure using BAPS indicated three major genetic clusters (Fig 2) that correspond with geographic origin, and a single outlier cluster that contained only four individuals with no apparent geographic pattern (two from RUM and two from IKR). The major genetic clusters were made up of samples from northwestern sites (KAP and RUM), southwestern sites (Serengeti ecosystem: GVR, MRT, FGT, NBS, MRB, GTR, IKR, KLM, MSN, MSS, NGK), and eastern sites (MNP, KIP, TSW, KIN, SHT, SHI, HND). NGU was placed in the eastern cluster in BAPs, but not in other analyses (see below). From here forward we refer to samples from western Kenya outside the Serengeti as the “northwest”, samples from within the Serengeti ecosystem as the “southwest”, and all other samples as the “east”. In description of these results for Kenya using KENTTEC recommended terminology, flies from the Lake Victoria basin fly belt (KAR and RUM) made up one of the three genetic clusters (northwest), flies from the Narok-Kajiado fly belt (plus all Tanzanian samples from the Serengeti ecosystem) made up another genetic cluster (southwest), and flies from the Mbeere-Meru fly belt, the Central Kenya fly belt, and the Coastal fly belt made up the third genetic cluster. The average probability of assignment (q-values) for the northwest was 0.97, the southwest (Serengeti ecosystem) was 0.97, and the east was 0.98 (Table 1; S7 Table). While most individuals were assigned to only one cluster associated with their region of origin, two individuals from both the northwest and southwest belonged to the outlier cluster, and eight individuals from both the southwest and east were genetically admixed with maximum q-values < 0.90 (Fig 2, S7 Table).

Results from the PCA fully supported BAPS, with strong separation between the west (northwest/southwest) and east apparent across PC 1 and 2 (accounting for 4.02% and 3.13% of the total variance, respectively), and separation between the northwest and southwest apparent along PC 4 (accounting for 2.08% of the total variance; S3 Fig). Results from STRUCTURE (S4 Fig) and DAPC (S5 Fig) largely agreed with BAPS with a single exception: These analyses placed NGU in the northwest rather than in the east, and indicated more admixture between the northwest and southwest (S4 and S5 Figs).

Pairwise F_ST_ between sampling sites averaged 0.123 and ranged widely from zero (NGK vs. MSS and KLM vs. MSN) to 0.312 (SHT vs. RUM) and were significant in 79% of pairs (Table 3; S8 Table) over a mean geographic distance of ~330 km (Fig 1).

**Table 3:** Pairwise genetic and geographic distance.

**(a)**	**KAP**		**RUM**	**GVR**		**MRT**		**FGT**	**NBS**		**MRB**	**GTR**		**IKR**		**KLM**	**MSN**		**MSS**		**NGK**
**KAP**			154.7	241.0		254.8		260.5	268.5		266.8	314.5		310.0		343.8	379.5		438.1		469.6
**RUM**	0.123			111.4		122.7		134.1	146.1		130.4	159.9		159.4		195.7	225.1		283.9		318.0
**GVR**	0.224		0.239			13.9		22.7	35.2		27.6	117.8		88.5		111.2	167.5		219.9		238.5
**MRT**	0.152		0.165	0.105				15.0	28.1		14.4	110.1		77.8		98.2	156.5		207.8		225.1
**FGT**	0.118		0.145	0.104		**0.010**			13.2		23.0	121.4		86.2		101.5	163.7		212.6		226.4
**NBS**	0.238		0.280	**0.006**		0.113		0.102			33.1	130.1		92.8		103.3	168.3		214.9		225.5
**MRB**	0.200		0.240	**0.012**		0.121		0.106	0.013			98.4		64.1		83.8	142.6		193.4		211.0
**GTR**	0.228		0.263	0.020		0.117		0.095	**0.001**		0.015			41.8		68.4	65.5		124.3		163.3
**IKR**	0.215		0.244	**0.011**		0.093		0.086	**0.006**		0.017	**0.008**				38.3	79.1		133.1		159.6
**KLM**	0.199		0.229	0.009		0.091		0.080	**0.009**		**0.010**	**0.010**		**0.007**			69.6		111.6		127.7
**MSN**	0.201		0.218	0.011		0.100		**0.090**	0.015		0.004	**0.013**		**0.009**		**-0.005**			58.8		101.5
**MSS**	0.217		0.239	0.017		0.113		0.097	**0.007**		0.013	**0.006**		**0.009**		**0.006**	**0.007**				54.8
**NGK**	0.241		0.259	0.016		0.116		0.108	0.011		0.014	0.014		0.012		0.014	0.013		**-0.002**		
**(b)**		**NGU**			**MNP**		**KIB**			**TSW**			**KIN**		**SHT**			**SHI**		**HND**	
**NGU**					217.4		217.6			400.0			472.9		449.8			524.1		305.7	
**MNP**		0.156					277.9			346.0			474.6		509.3			518.0		389.5	
**KIB**		0.170			0.017					74.0			214.3		258.7			276.2		317.4	
**TSW**		0.175			0.014		0.023						140.7		186.5			205.9		303.6	
**KIN**		0.136			0.030		0.017			0.033					50.3			76.5		307.7	
**SHT**		0.163			0.077		0.082			0.093			0.088					28.5		300.0	
**SHI**		0.158			0.028		**0.006**			0.033			**0.010**		0.052					285.3	
**HND**		0.161			0.026		**0.003**			0.024			**0.006**		0.082			**0.003**			

Pairwise F_ST_ and geographic distance (below and above the diagonal, respectively) between site pairs within the **(a)** northwest and southwest (shaded grey), and **(b)** east. Pairwise F_ST_ was computed in Arlequin [44] based on Weir and Cockerham 1984 [65]. Non-significant F_ST_ values (p > 0.05) are in bold.

**Fig 3:**
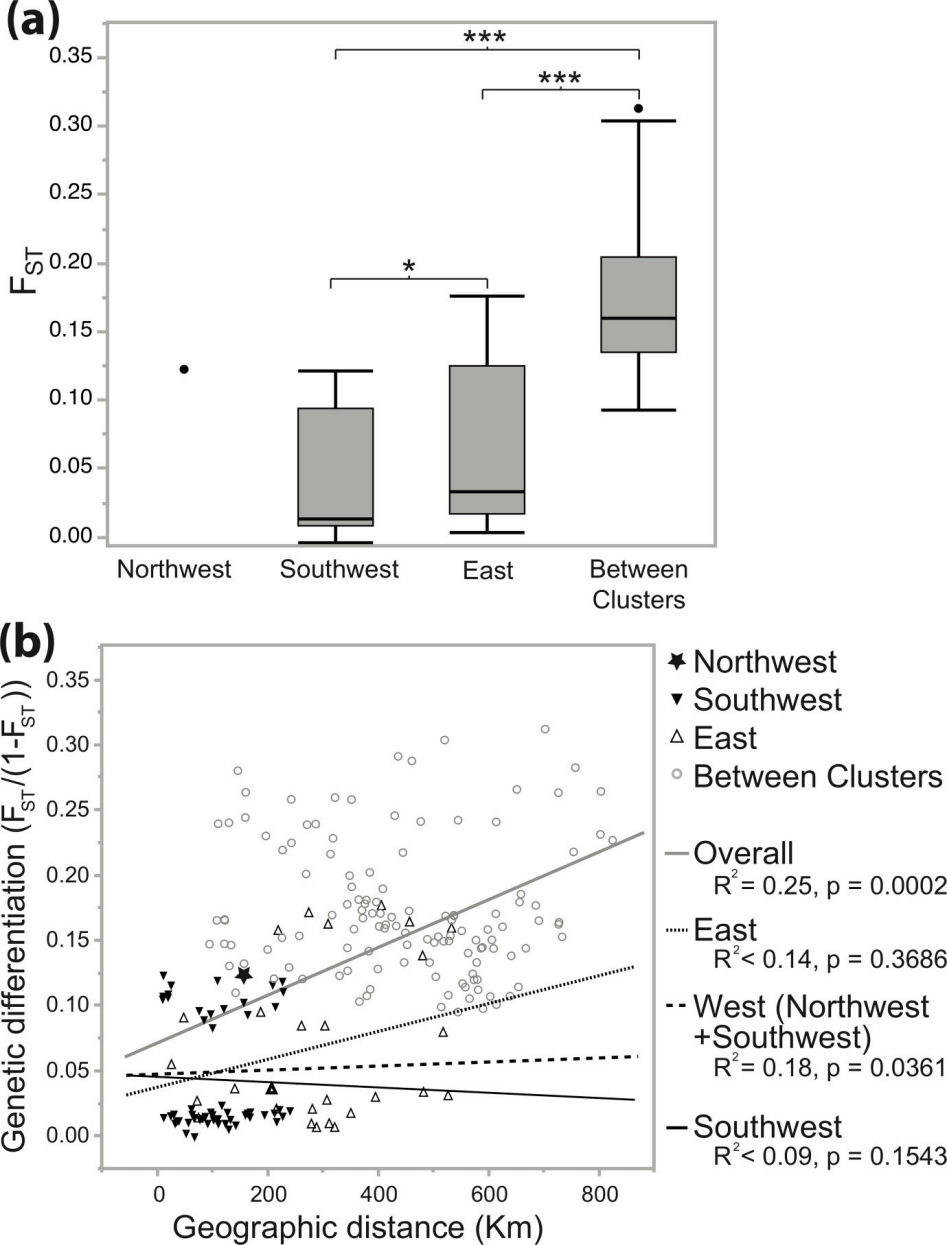
Comparison of F_ST_ among and between clusters, and relationship between F_ST_ and geographic distance. **(a)** Genetic differentiation (F_ST_) computed in Arlequin [44] based on Weir and Cockerham 1984 [65] within and among clusters. Box plots show the mean, 1^st^ and 3^rd^ quartile, 95% quantiles (whiskers) and outliers (dots). Student’s t-tests indicated that average F_ST_ was significantly lower in the Southwest than the East (p = 0.0273, marked *), and significantly higher between-cluster comparisons than in the southwest or east (p < 0.0001, marked ***). **(b)** Genetic versus geographic distance using F_ST_/(1- F_ST_) to correct for finite population sizes [66] plotted for the northwest (star), southwest (downward pointing triangles), east (upward pointing triangles), and between clusters (grey circles), with linear line of best fit with R^2^ and p-values for Mantel tests for isolation by distance [66,68] performed in the “isolation by distance” web service v3.23 [67].

The northwest only contained one pair of sampling sites (KAP and RUM) separated by ~155 km with a significant F_ST_ of 0.123 (Table 3A), and could not be included in any statistical tests. The southwest had an average F_ST_ of 0.040 over a mean geographic distance of ~111 km (Table 3A). 62% of southwest F_ST_ estimates were significant. The east had significantly higher F_ST_ than the southwest (Student’s t-test p = 0.0273; Fig 3A), averaging 0.067 over a mean geographic distance of ~282 km, which was not surprising given the larger geographic distances separating sites (Table 3A). 82% of east F_ST_ estimates were significant. There was also significantly higher genetic differentiation between clusters (average F_ST_ = 0.123) than among sites in the southwest or east clusters (Student’s t-test p < 0.0001; Fig 3A).

There was significant isolation by distance across each genetic break: Across the east/west genetic break (overall: p = 0.0002), and across the northwest/southwest genetic break (west: p = 0.0361). In contrast, there was no significant isolation by distance within any of the genetic clusters (Fig 3B). Indeed, genetic and geographic distance in the southwest and east were remarkably unlinked. In the southwest, the pair of sampling sites with the lowest F_ST_ (KLM and MSN: F_ST_ = -0.005) were separated by a full 69.6 km, and the pair of sampling sites with the highest F_ST_ (MRT and MRB: F_ST_ = 0.121) were separated by only 14.4 km (Table 3A). In the east, the pair of sampling sites with the lowest F_ST_ (KIB and HND: F_ST_ = 0.003) were separated by a full 303.6 km, and the pair of sampling sites with the highest F_ST_ (KIN and NGU: F_ST_ = 0.175) were separated by 400 km, a distance shorter than the maximum of 509.3 km separating SHT and MNP (Table 3B).

### Migration

**Fig 4:**
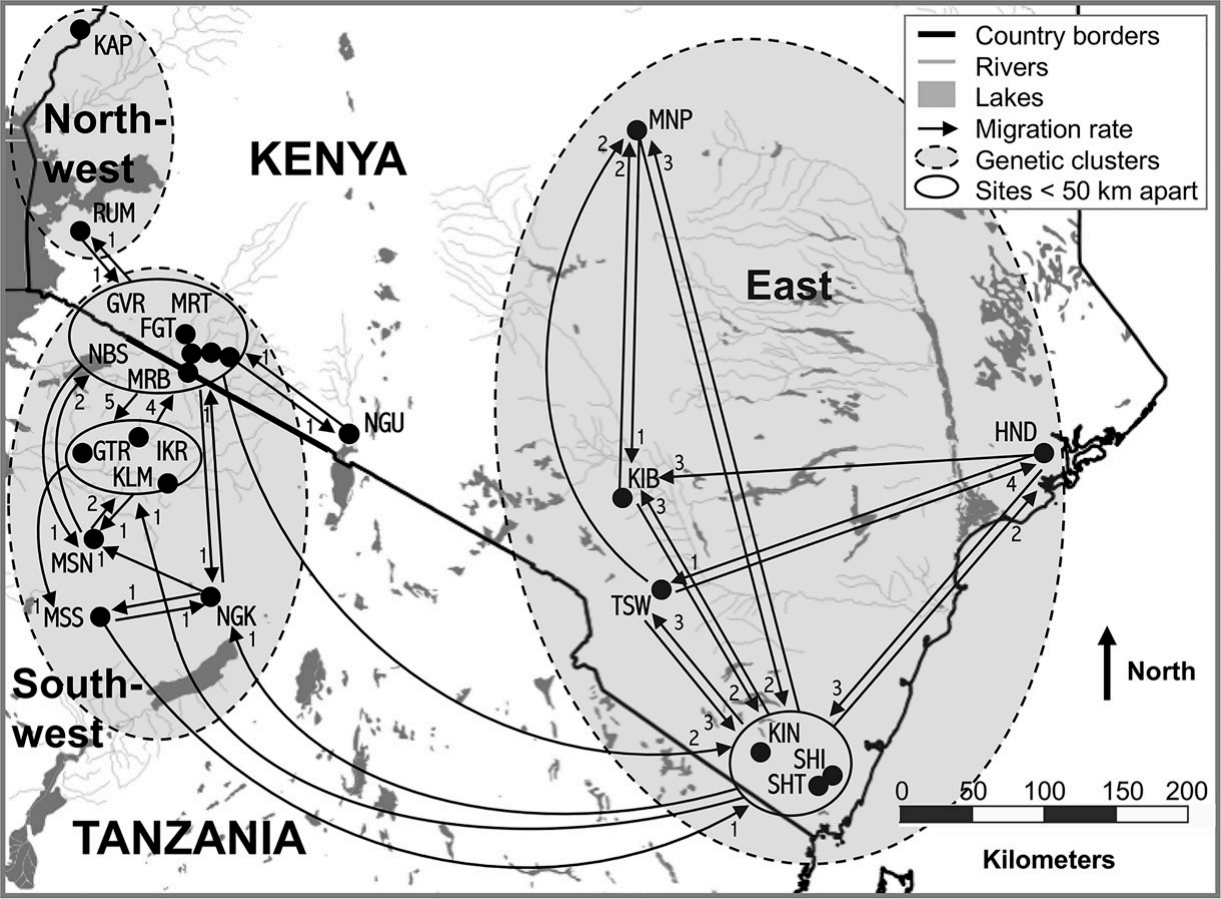
First generation migrants among sampling sites. Migrants detected using the software GENECLASS [70] indicated with arrows pointed in the direction of movement. Sites were grouped together if less than 50 km apart. Each dot represents a sampling site labeled with site codes (Table 1). The dashed outlines denote the three genetic clusters identified in BAPS v 6 [55,56]. The map was created in QGIS v2.12.1 (August 2017; http://qgis.osgeo.org) with free and publicly available data available from DIVA-GIS (August 2017; http://www.diva-gis.org).

With the relatively conservative p-value cutoff (0.05) designed to identify all potential first-generation migrants, GENECLASS identified 83 migrants, with zero migrants within the northwest, 38 migrants within the southwest, and 38 migrants within the east (Fig 4, S9 Table). The southwest had the highest exchange between any two sites between MRT and FGT (8 migrants; S9 Table), two sites separated by only 15 km in the Kenyan part of the Serengeti ecosystem. The east had migration over both large and small geographic scales, as we detected migrants between sites separated by 278 km (MNP and KIB) to only 20 km (SHI and SHT; Table 3). There were 9 between-cluster migrants detected, one in each direction between the northwest and southwest, and seven between the southwest and east (four from the southwest that were detected in the east, and three from the east that were detected in the southwest; S9 Table). There was no statistical difference in rate of migration between the sexes (43 females *versus* 40 males; S9 Table). Only two migration events within the Serengeti ecosystem (from IKR to GTR, and from IKR to MRB) were significant after correcting for multiple testing (S9 Table).

### Population history modeled by ABC

Prior checking indicated non-significant differences between the most summary statistics calculated for simulated and observed mtDNA and microsatellite data under the winning scenario (S3 File; S4 File, respectively), and some overlap in results of the PCA of the simulated and observed summary statistics (S6 Fig). However, there was high posterior predictive error in both Analysis 1 (S3 Table), suggesting lack of power to reliably identify the correct scenario. These results suggest that neither of the datasets (mtDNA or microsatellites) provided the power needed to accurately identify the true pattern of ancestry (Analysis 1). Furthermore, it is likely that the microsatellite dataset could not provide accurate estimates of time of divergence (Analysis 2) because microsatellites generally have fast mutation rates that make them inappropriate to estimate timing of splits on the order of millions of years that was indicated in the mtDNA analysis (S7 Fig).

There were, however, consistent indications from the mtDNA Analysis 2 that scenarios with variable N_e_ (Scenarios 1b, 2b, 3b, and 4b) were supported over scenarios with constant N_e_ (Scenarios 1b, 2b, 3b, and 4b; S3 Table). Parameter estimates indicated that the timing of divergence between the northwest and southwest corresponded to no divergence (i.e. mode of estimate of t1 = 0), that there was ancient divergence on the order of millions of years between the west (northwest and southwest) and east (S7 Fig), and that bottlenecks in the northwest and southwest may have occurred within the last 2–100 years. Results from the microsatellite analysis generally agreed with the mtDNA results but we also found several differences. Contrary to mtDNA results (S3 File), microsatellite analysis indicated that divergence between the northwest and southwest (t1) occurred between 1,000 and 10 million years ago (S7 Fig). Additionally, microsatellite results under Scenario 3 supported constant over variable N_e_, a result contrary to results from all mtDNA analyses and microsatellite analysis under Scenarios 1, 2, and 4 (S1 Fig; S3 Table).

## Discussion

### Genetic diversity

Genetic diversity estimates from the northwest and southwest had slightly lower mean allelic richness values as compared to the east. This difference in genetic diversity could reflect differences in ecology, and/or differences in anthropogenic history including habitat destruction, grazing pressures and vector control. Ecological differences include less seasonality and larger areas of undisturbed habitat within river basins in the east (see discussion of genetic connectivity below). Differences in anthropogenic disturbance likely played a role in shaping patterns of genetic diversity. Urbanization, habitat destruction, agricultural activity, high grazing pressures, and a history of tsetse control measures [28] in western Kenya [4–6] in the 1980s and 2000s [89–91] driven by the presence of HAT and recurring outbreaks of AAT may all have played a role in reduced population sizes and thus reduced genetic diversity of *G*. *pallidipes* in the northwest and southwest as compared to the east. Lower estimates of N_e_ in the northwest and southwest (Table 2) and indications of recent population reductions in the ABC results (i.e. mode of estimates of date of bottleneck db_NW_ and db_SW_ = 2–100 years ago; S7 Fig; S3 Table), both lend further support for the hypothesis that anthropogenic disturbance has reduced *G*. *pallidipes* population sizes in western Kenya within the last 100 years.

We detected 49 occurrences of significant deviation from HWE (S4 Table), this could be because of a deficit in heterozygotes leading to positive and significant F_IS_ values and suggest that individuals in our study may be related (~ 10% of individuals; Table 2), or that there may be a history of inbreeding. However, estimates of deviations from HWE after excluding putative relatives (S6 Table) were very similar to the estimates made with the full dataset, which was similar to the result found by [26], and favors inbreeding as an explanation for deviations from HWE. One possibility is that the signal of high relatedness could be a result of inbreeding caused by life history traits common to the genus, *Glossina*. For example, viviparity [80] mandates that there is only one offspring per reproductive cycle, and only ~ three offspring in the lifetime of a female. This results in small effective population sizes and a high probability of inbreeding when close relatives encountering one another during reproduction. Another factor could be the short distance of average dispersal of tsetse flies by flight of < 2 km [92–94] et ref 92 Rodgers et, which could also increase the probability of relatives encountering one another during reproduction.

We did not detect any signals of genetic bottlenecks (Table 2) using TPM and IAM models as well as using mode-shift indicator test. Previous work by Ciosi et al., 2014 [82] identified a genetic bottleneck in KAP and this was attributed to previous tsetse control efforts that had been carried out in the area [89]. The discrepancy between the two results could be due to the timing of the sampling in the two studies and the limited sensitivity of the BOTTLENECK approach to detect bottlenecks in the distant past. Ciosi et al., 2014 [82] used samples that were collected in the year 2000, just a few years after tsetse control measures were enforced, while samples for this study were collected in the year 2016, a difference of ~128 generations. It could be that during this time span the population could have recovered from the bottleneck effect.

### Population structure

Genetic clustering results, while largely agreeing with findings from a previous analysis on a narrow transect along the southern border of Kenya [26], provide a more clear definition of the three genetic clusters (northwest, southwest, and east) and their boundaries. All clustering methods (BAPS, STRUCTURE, DAPC, PCA) identified a distinct genetic break between sampling sites to the east and west of the Ethiopian Rift, and a genetic break between the northwest (KAR and RUM) and the Serengeti ecosystem in the southwest (Fig 2, S3–S5 Figs). The membership of NGU to the cluster east or west of the Ethiopian Rift was different in BAPS than in the other analyses (Fig 2, S3–S5 Figs). We favor the BAPS results here because this method accounts for uneven spatial sampling (spatial autocorrelation) [55,56]. However, it should be noted that none of the analyses used could also correct for the possibility that the genetic breaks were caused by isolation by distance rather than genetic divergence across a geographic barrier [95] and this remains a possibility. Indeed, the ABC analysis suggests that the northwest/southwest genetic break may not represent genetic divergence across a geographic break because there is some evidence that the timing of this population split was contemporary (see below). Nonetheless, the fact that there is no signal of isolation by distance within genetic clusters argues that the cause of the genetic breaks was not uniform isolation by distance. Instead, patterns of divergence and Hardy-Weinberg equilibrium identified in the BAPS and STRUCTURE analyses suggest that the three genetic clusters identified may have unique population dynamics [35, 36, 52].

### Genetic differentiation and migration

In general, most pairs of sampling sites were significantly differentiated despite being geographically separated by as little as ~13 km (Table 3), and there was an overall pattern of isolation by distance (Fig 3B). However, there was no pattern of isolation by distance within genetic clusters, and each region appeared to have unique patterns of genetic connectivity. In the southwest, there was surprisingly high genetic differentiation across short geographic distances, with pairs of sites separated by only 13.2 km (NBS and FGT), 13.9 km (GVR and MRT), and 14.4 km (MRT and MRB) displayed highly significant genetic differentiation with FST values ~0.1 (Table 3). Conversely, there were low levels of genetic differentiation among sites centrally located within the southwest. This indicates high differentiation in the northern extent of the Serengeti ecosystem, and low genetic differentiation in the central region of the Serengeti ecosystem regardless of geographic distance. Low genetic differentiation in the central region of the Serengeti could be caused by habitat connectivity during the wet season, which could facilitate fly dispersal and thus gene flow over multiple generations [96–99].

In the east, there were high levels of genetic connectivity even across distances greater than 300 km, with F_ST_ values ranging from a low of 0.003 between HND and KIB separated by 317.4 km, to a high of 0.024 between TSW and HND separated by 303.6 km (Table 3). The east had only slightly higher F_ST_ estimates than the southwest (0.067 *vs* 0.040), and this was over much larger average geographic distances (110.6 km *vs* 282.2 km; Fig 3; Table 3). This implies greater genetic connectivity in general in the east (Table 3), which aligns with greater genetic diversity (Table 1), and higher migration rates (Fig 4), both patterns noted in previous studies [26][98]. Notably, there was surprisingly low genetic differentiation across large geographic distances (Fig 1) among sites in the Athi-Galana-Sabiki river basin separated by 74 km (KIB and TSW) and sites in the Tana river basin separated by 389.5 km (HND and MNP; Table 3). These low levels of genetic differentiation imply *G*. *pallidipes* gene flow is high within the Athi-Galana-Sabaki River basin and between the Tana and the Athi-Galana-Sabaki river basins, and highlights a potential major role of large river basins in driving patterns of gene flow in *G*. *pallidipes*.

High genetic connectivity in the east, especially among sites within river basins, could reflect the ecology of the region, and/or the anthropogenic history of the region. Ecologically, high connectivity could be driven by low seasonal variation in water availability in the coastal forest habitat that allows for more continuous high population densities. This is supported by the N_e_ estimates and the ABC results, which indicated larger population sizes in the east and more constant population size throughout evolutionary history. Additionally, habitat connectivity within river basins, which are larger in the east than in the west, and with other *G*. *pallidipes* populations that exist in a continuous distribution from northeast Tanzania to southern Somalia [100–102], could both contribute to more stable population sizes, higher genetic connectivity, and higher genetic diversity in the east than in the northwest and southwest. Regarding anthropogenic history, lower levels of urbanization, livestock density, and HAT disease risk in eastern Kenya [4–6] has resulted in a lower level of habitat alteration and vector control activity, which may have contributed to more continuous and stable tsetse populations, and could help explain the higher genetic diversity found in the east.

### Population history modeled by ABC

Results from the ABC analysis were difficult to interpret because of high predictive error of ~0.7 in the analysis designed to distinguish the pattern of ancestry (Analysis 1: S3 Table), and inconsistency in parameter estimates in the analysis designed to refine estimates of population size and timing of population splits (Analysis 2: S7 Fig).

Even so, there were some consistent patterns that emerged from the mtDNA analysis that showed minimal divergence between the northwest and southwest, deep divergence between the east and west, and population size fluctuations in the northwest and southwest. The winning scenarios in Analysis 2 always included *G*. *pallidipes* population size fluctuations in the northwest and southwest during the last century (S3 Table), and negligible divergence between the northwest and southwest (S7 Fig). This suggests that the genetic break between the northwest and southwest perhaps represents isolation by distance across a geographic gap in sampling. The signal of a genetic break could have been accentuated by recent population size fluctuations in these regions, which would have increased differences in genotype frequencies and Hardy-Weinberg disequilibrium between samples from the two regions [35,36]. In contrast, ABC results suggest a deep divergence time on the order of millions of years between the east and west (S7 Fig). This opens the possibility that there are reproductive barriers between these two genetic clusters, but should be confirmed with further research that provides evidence of divergence beyond isolation by distance. Existence of reproductive barriers would mean that even when flies migrate between the east and west, as detected in our migration analysis (Fig 4), reproductive success would be low in migrated individuals and would pose a low risk of providing population augmentation or the introduction of novel *G*. *pallidipes* genetic variation in the receiving population.

If divergence is as old as a million years, reproductive barriers could have accumulated. Reproductive barriers would reduce the risk of population augmentation from populations from a neighboring genetic cluster. However, it would not remove the threat of re-establishment after local eradication from populations from a neighboring genetic clusters, if the ecological needs of the invading population were met. Future research should assess the levels of interbreeding among the three genetic clusters and characterize any reproductive barriers that may exist to determine the level of threat posed by reinvasion across the boundaries between genetic clusters.

### Conclusions and recommendations for effective vector control strategies

Our findings provide an understanding on the levels and patterns of genetic diversity, differentiation, gene flow, and population dynamics among and within *G*. *pallidipes* populations sampled from western and coastal Kenya as well as the Serengeti ecosystem in Tanzania. Results from the multiple analyses indicate that there is non-random mating across the range, and that *G*. *pallidipes* populations are partitioned into three clusters (northwest, southwest, and east), with *G*. *pallidipes* genotypes fitting expectations of Hardy-Weinberg equilibrium only when separated into these three groups. Along with significant population differentiation at multiple scales and lack of isolation by distance within genetic clusters, these results suggest that the major population dynamics such as the population density, the distance of average dispersal, and disease transmission dynamics will be unique to each genetic cluster. Even if the genetic break between the northwest and southwest was caused by isolation by distance rather than a geographic barrier, these regions are ecologically and epidemiologically different because of the conservation status of the Serengeti ecosystem (i.e. there are different large mammals present, cattle grazing patterns, and human visitation rates) and so should be treated differently during tsetse fly control campaigns.

Using KENTTEC recommended terminology, these results indicate that the Lake Victoria tsetse belt and the Narok-Kajiado fly belt are in separate genetic clusters, but that the three tsetse belts in the east (Mbeere-Meru fly belt, the Central Kenya fly belt, and the Coastal fly belt) have high genetic connectivity in *G*. *pallidipes* and should be considered as a single *G*. *pallidipes* population. The results imply that in eastern Kenya for all three KENTTEC terminology fly belts (Coastal, Mbeere-Meru, and Central Kenya fly belts), *G*. *pallidipes* eradication may likely never be feasible, and that suppression rather than eradication would be a more realistic target. Results also indicate evidence of infrequent migration between the clusters, which could pose a reinvasion threat after local eradication, if it were to be successful in the northwest or southwest.

N_e_ estimates and ABC results indicated that the northwest and southwest have gone through a recent population size reduction and currently have lower N_e_ and less genetic variation than populations in the east. Results also indicated relatively small N_e_ (<100) in a subset of *G*. *pallidipes* (KAP in the northwest, IKR, MRB, GTR and MSN in the southwest, KIB and MNP in the east), suggesting that novel vector control methods may be feasible in these regions. There is evidence from disease transmission models that novel control methods such as inundation and replacement of natural populations with sterile males, or genetically/endosymbiont modified flies (e.g., replacement with artificially selected low vector competence individuals as suggested by Powell & Tabachnick [103], or replacement with flies with modified endosymbionts as suggested by Aksoy ([46]) are more effective in small populations [46,103]. On the other hand, small N_e_ suggests localized dispersal and breeding, and means that genetic modification will require many local releases that target spatially separated populations across a larger target area [103]. Thus, successful replacement may only be feasible in the subset of populations with small N_e_ that are also distributed over a small geographic area, such as the population in the northwest (i.e. in the RUM region).

Taken together, results suggest that models of transmission dynamics should consider the northwest, southwest, and east separately, and that tsetse control strategies should be designed as a coordinated effort for each genetic cluster. Specifically, eradication will likely never be feasible in the in eastern Kenya for all three KENTTEC terminology fly belts (Coastal, Mbeere-Meru, and Central Kenya fly belts), while there is potential for success of novel vector control methods that require inundation and replacement of natural populations in geographically isolated populations with small N_e_, such as those found in the northwest. Furthermore, our data suggest that infrequent long-range migration events do occur even between distinct populations separated by more than 200 km (Fig 4), underscoring the need for active monitoring of fly movement to minimize risk of augmentation from neighboring populations and reestablishment after successful local eradication. Further studies to investigate reproductive barriers among genetic clusters are needed to identify the risk of population subsidy and/or replacement after control efforts. Likewise, further studies to investigate the distributions of populations with small N_e_ with spatial modeling are needed to identify isolated populations where novel control techniques such as genetically modifying vector populations can be tested and developed further. Finally, further studies to resolve demographic and genetic connectivity patterns in the northwest are needed, as we had sparse sampling in this region and results indicated unique population connectivity, genetic variation, and demographic patterns.

## Supporting information

S1 Fig Competing scenarios considered in ABC models of population history. Alternative scenarios **(a)** without fluctuating population sizes (Scenarios 1a, 2a, 3a, 4a), considered in Analysis 1 designed to identify the most likely ancestral lineage, and **(b)** with fluctuating population sizes in the northwest and southwest (Scenarios 1b, 2b, 3b, 4b) considered in Analysis 2 to further refine estimates of timing and N_e_ for each of the genetic clusters. Priors were based on published estimates and the geologic record (S3 Table).
(DOCX)

S2 Fig Principal components analysis of genetic variation. Results of the principal components analysis conducted with the "adegenet" package v2.1.1 (Jombart et al., 2018) in R Studio v1.1.383, showing the variance found in the three principal components that display separation among the major clusters detected in BAPS v 6 [55,56]. These three components were PC 1, 2, and 4, and explained 4.02%, 3.13%, and 2.08% of the variance in microsatellite genotypes, respectively. Individuals are represented by dots color coded by cluster to match S1 Fig (northwest = orange, southwest = blue, east = purple, outlier cluster = yellow, and admixed individuals = grey).
(DOCX)

S3 Fig STRUCTURE results. STRUCTURE results for K = 1–10. Each bar represents a single fly with the proportion of colors representing the Bayesian probability of assignment (q-value) of an individual. Black lines separate sampling sites.
(DOCX)

S4 Fig Discriminant analysis of principle component (DAPC). DAPC based on *G*. *pallidipes* microsatellite data for 21 sampling sites, completed in the R (R Development core team) using the “adegenet” package [63] with 40 principle components. Individuals are represented by dots linked by a line to the centroid and encompassed by 95% confidence intervals. Colors represent assignment to genetic cluster from the BAPS v 6 [55,56] analysis (orange = northwest, blue = southwest, purple = east). PCA eigenvalues represent variance explained by principle components, with the components included in the analysis shaded dark grey.
(DOCX)

S5 Fig Mitochondrial DNA sequences haplotype network. TCS haplotype network where haplotypes are represented by circles that are sized proportionally to frequency and shaded with the genetic cluster they were chosen to represent in the ABC analysis. Hashes along the branches of the network represent a single nucleotide change (one inferred mutation), and black dots represent unsampled haplotypes.
(DOCX)

S6 Fig Analysis of reliability of ABC results. Principal components analysis (PCA) from **(a)** mtDNA under scenarios without fluctuating population sizes (Scenarios 1a, 2a, 3a, 4a), **(b)** mtDNA based results under scenarios with fluctuating population sizes (Scenarios 1b, 2b, 3b, 4b), **(c)** microsatellites based results under scenarios without fluctuating population sizes (Scenarios 1a, 2a, 3a, 4a), **(b)** microsatellite based results under scenarios with fluctuating population sizes (Scenarios 1b, 2b, 3b, 4b). Results from different scenarios are colored as indicated in the legend. ABC analyses was performed in DIYABC v2.0.4 [74].
(DOCX)

S7 Fig Parameter estimates from ABC analysis. **Mode of** DIYABC v2.0.4 [74] parameter estimates from the winning scenarios of Analysis 2 (Scenarios 1b, 2b, 3b, 4b) in green, red, blue, and pink, respectively, including estimates of **(a)** population size from the mtDNA analysis **(b)** timing of simulated events from the mtDNA analysis, **(c)** population size from the microsatellite analysis, and **(d)** timing of simulated events from the microsatellite analysis plotted on a log scale to make all estimates visible in a single image. Population size estimates are presented for the northwest after a population bottleneck (NW_b_), the southwest after a population bottleneck (SW_b_), ancestral northwest (NW), ancestral southwest (SW), and the east (E). Timing estimates are presented for the date of bottleneck for the northwest (db_NW_), date of bottleneck for the southwest (db_SW_), the population split between the northwest and southwest (t1), and the population split between the west and east (t2).
(DOCX)

S1 Table Fourteen sampling sites in Kenya where tsetse flies were not caught during the study. Site name, site ID, county, latitude, longitude and sampling data of the 14 locations that did not have any flies during field collections despite past collection records that indicated the presence of *G*. *pallidipes*.
(DOCX)

S2 Table Information on microsatellite loci used in analyses. The table shows loci names in the first column followed by fluorescent dye used for each locus, DNA sequences of the forward and reverse primers, size range of alleles in base pairs (bp), repeat motif length in base pairs and publication reference for primer design.
(DOCX)

S3 Table ABC priors, and posterior probabilities of competing scenarios. ABC modeling was done using DIYABC v2.0.4 [74]. Panel **(a)** displays the prior minimum (min) and maximum (max) values of the priors used in the simulations and the scenarios these priors applied to. Panel **(b)** and **(c)** display results for the mtDNA and microsatellite based ABC analyses, respectively. Results displayed include the relative posterior probability of the scenario tested using the weighted logistic regression method described by [70], the lower 95% confidence interval of the posterior probability (CI), the upper CI, and the posterior predictive error (frequency of accepting a scenario other than the true scenario in 1000 runs of model checking with simulated data). All time priors (t1, t2) and the timing of the bottlenecks are displayed in years assuming a generation time of 5 per year.
(DOCX)

S4 Table F_IS_ values after testing for deviation from Hardy Weinberg equilibrium (HWE) using. Bolded F_IS_ values were considered significant after Benjamini-Hochberg correction for multiple testing.
(DOCX)

S5 Table Results of pairwise tests for LD estimated using *Genepop v4.6* [41] showing chi-squared (χ2) distribution per locus pair, degrees of freedom (df), and original p-values for the test for significance and p- values after Benjamini-Hochberg correction for multiple testing. (DOCX)

S6 Table Summary statistics with a subset of the data without closely related individuals from each site, number of samples remaining (N), mean allelic richness across loci (AR), observed (HO) and expected heterozygosity (HE), inbreeding coefficient (FIS), and FIS p-values. (DOCX)

S7 Table Individual assignments to genetic clusters. Probability of assignment (q-value) for individuals to each of the four clusters identified in BAPS v 6 [55,56]. Admixed individuals (< 0.9 assignment probability to any one cluster) and those assigned to the outlier cluster are shown in bold.
(DOCX)

S8 Table Genetic and geographic distance of between-cluster pairs. This table shows **(a)** pairwise genetic differentiation (F_ST_) and **(b)** geographic distance (in km) among pairs that were not included in Table 3 in the main text. Pairwise F_ST_ was computed in Arlequin [44] based on Weir and Cockerham 1984 [65]. Significant values (p > 0.05) are denoted in bold.
(DOCX)

S9 Table First generation migrants among sampling sites. Home site, sample ID, sex of the migrant, inferred origin of the migrant, and p-value of the test of migrants detected using GENECLASS [70] **(a)** within the southwest, **(b)** within the east, **(c)** between the northwest and southwest, and **(d)** between the southwest and east. Marginally significant after Benjamini-Hochberg correction for multiple testing (corrected p-value = 0.0308) are marked with *.
(DOCX)

S1 File Binning rules used in the program GENEMARKER v2.4.0 (Soft Genetics, USA). We strongly caution that these rules may only be valid for PCR amplifications and genotype calls made with samples processed on the same Mastercycler Pro Thermocycler (Eppendorf, Germany) and ABI 3730xL Automated Sequencer (Life Technologies, USA) at the DNA Analysis Facility on Science Hill at Yale University (http://dna-analysis.yale.edu/). Instead of relying on these binning rules, we recommend that researchers request and use DNA from the same individuals as controls to calibrate future studies carried out on different equipment.
(TXT)

S2 File Detailed Approximate Bayesian Computation (ABC) methods and results. Methods of the mitochondrial DNA sequencing done for ABC analysis, and the ABC scenarios used are described. Basic results of the mitochondrial data included, and ABC analysis are reported.
(DOCX)

S3 File Details of mitochondrial DNA sequence based Approximate Bayesian Computation (ABC) results. DIYABC v2.0.4 [74] output for the mtDNA-based analysis from **(a)** Analysis 1 comparing Scenarios 1a, 2a, 3a, and 4a, **(b)** Analysis 2 comparing Scenarios 1a and 1b, **(c)** Analysis 2 comparing Scenarios 2a and 2b, **(d)** Analysis 2 comparing Scenarios 3a and 3b, and **(e)** Analysis 2 comparing Scenarios 4a and 4b. Output includes prior checking, posterior probability comparison of scenarios, and parameter estimation. The details of the scenarios are in S1 Fig.
(TXT)

S4 File Details of microsatellite-based Approximate Bayesian Computation (ABC) results. DIYABC v2.0.4 [74] output for the microsatellite-based analysis from **(a)** Analysis 1 comparing Scenarios 1a, 2a, 3a, and 4a, **(b)** Analysis 2 comparing Scenarios 1a and 1b, **(c)** Analysis 2 comparing Scenarios 2a and 2b, **(d)** Analysis 2 comparing Scenarios 3a and 3b, and **(e)** Analysis 2 comparing Scenarios 4a and 4b. Output includes prior checking, posterior probability comparison of scenarios, and parameter estimation. The details of the scenarios are in S1 Fig.
(TXT)
