# Supplementary figures and images for: Phylogeography and population structure of the tsetse fly Glossina pallidipes in Kenya and the Serengeti ecosystem
Source: PLoS Negl Trop Dis. 2020 Feb 24;14(2):e0007855. doi: 10.1371/journal.pntd.0007855 (PMC7058365; doi:10.1371/journal.pntd.0007855)

**
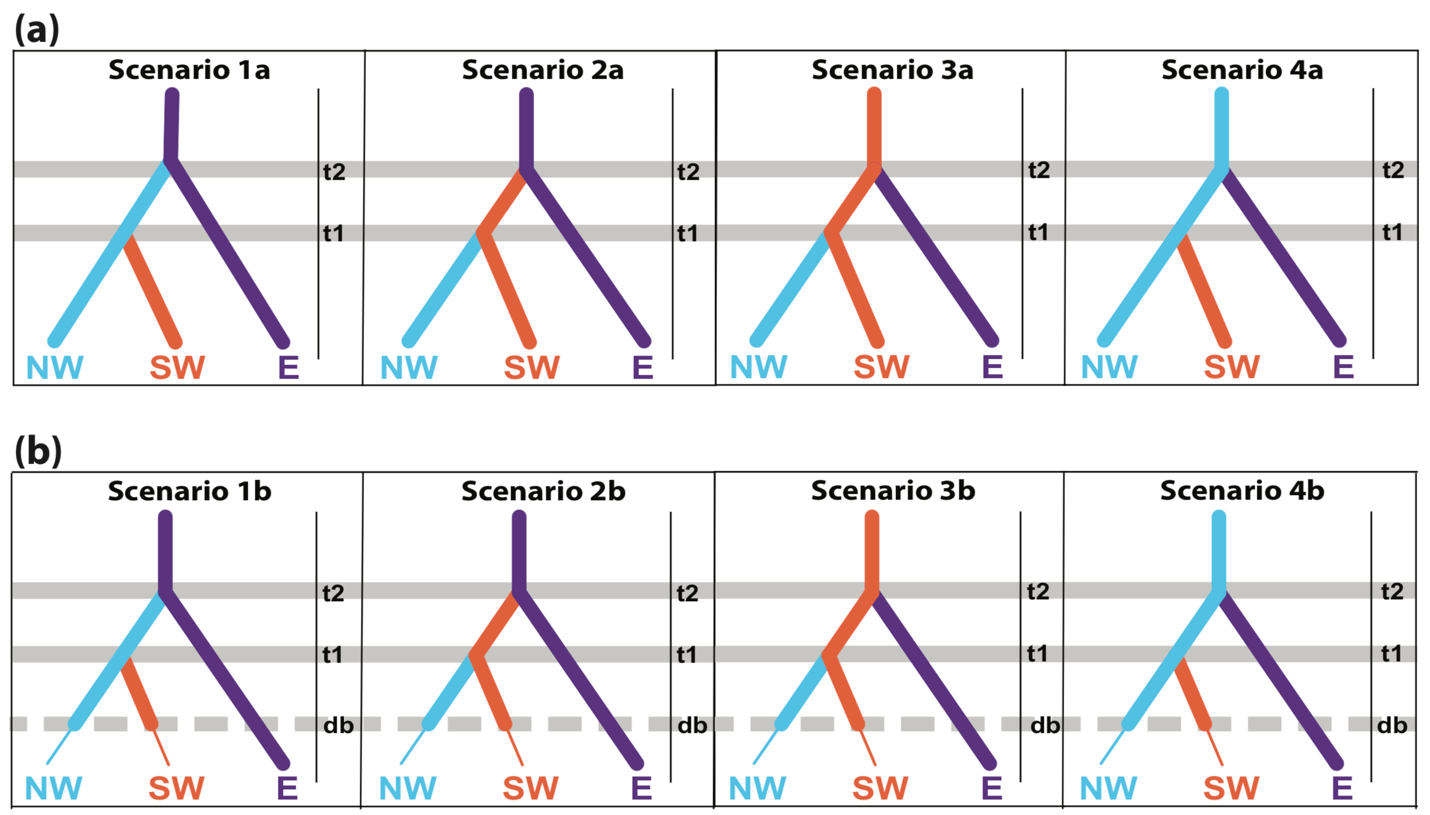
**

**S1 Fig.**

Supplement: S1 Fig — Alternative scenarios (a) without fluctuating population sizes (Scenarios 1a, 2a, 3a, 4a), considered in Analysis 1 designed to identify the most likely ancestral lineage, and (b) with fluctuating population sizes in the northwest and southwest (Scenarios 1b, 2b, 3b, 4b) considered in Analysis 2 to further refine estimates of timing and Ne for each of the genetic clusters. Priors were based on published estimates and the geologic record (S3 Table). (DOCX) [file pntd.0007855.s001.docx]

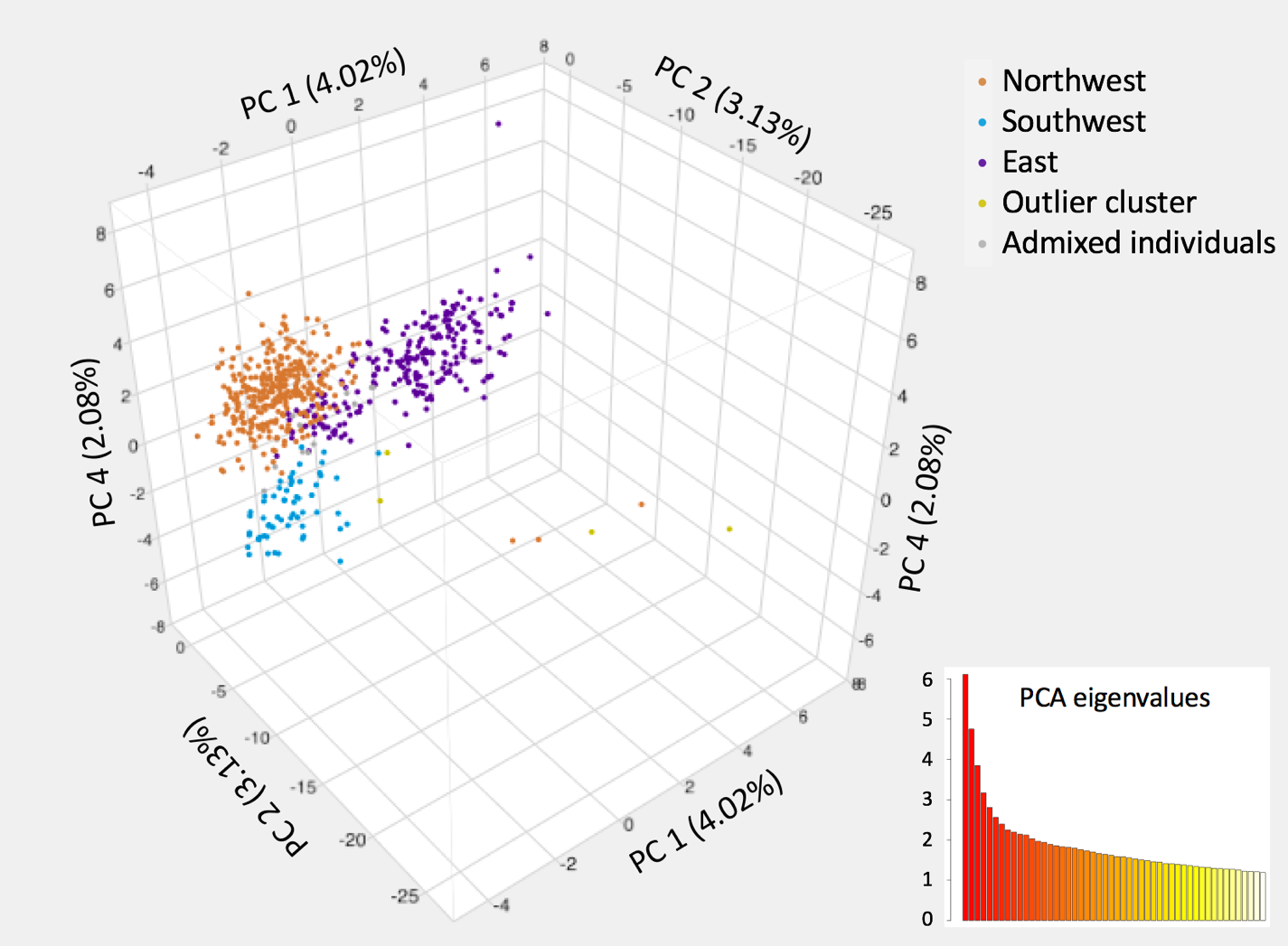


**S2 Fig.**

Supplement: S2 Fig — Results of the principal components analysis conducted with the "adegenet" package v2.1.1 (Jombart et al., 2018) in R Studio v1.1.383, showing the variance found in the three principal components that display separation among the major clusters detected in BAPS v 6 [55,56]. These three components were PC 1, 2, and 4, and explained 4.02%, 3.13%, and 2.08% of the variance in microsatellite genotypes, respectively. Individuals are represented by dots color coded by cluster to match S1 Fig (northwest = orange, southwest = blue, east = purple, outlier cluster = yellow, and admixed individuals = grey). (DOCX) [file pntd.0007855.s002.docx]

**
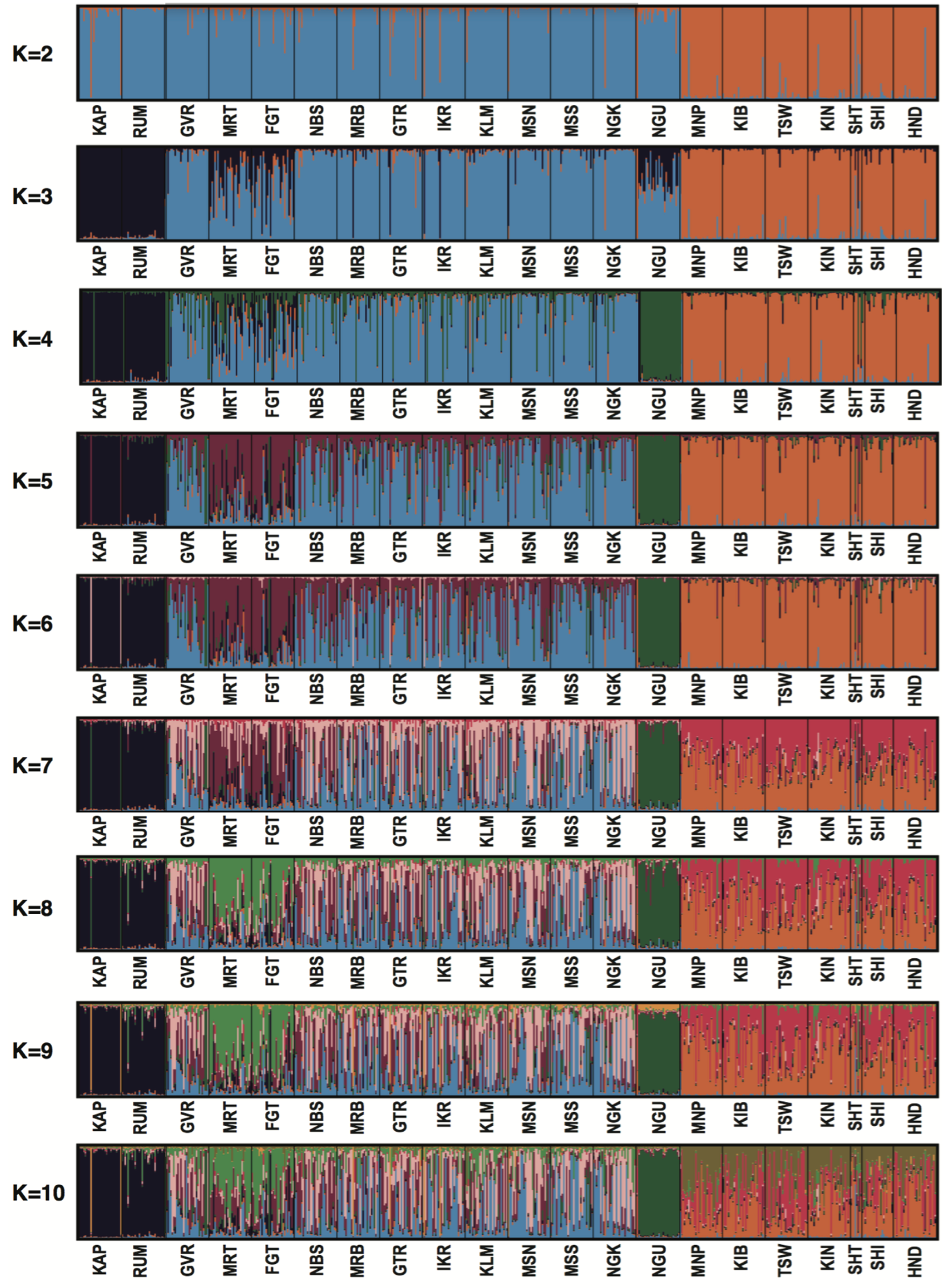
S3 Fig.**

Supplement: S3 Fig — STRUCTURE results for K = 1–10. Each bar represents a single fly with the proportion of colors representing the Bayesian probability of assignment (q-value) of an individual. Black lines separate sampling sites. (DOCX) [file pntd.0007855.s003.docx]

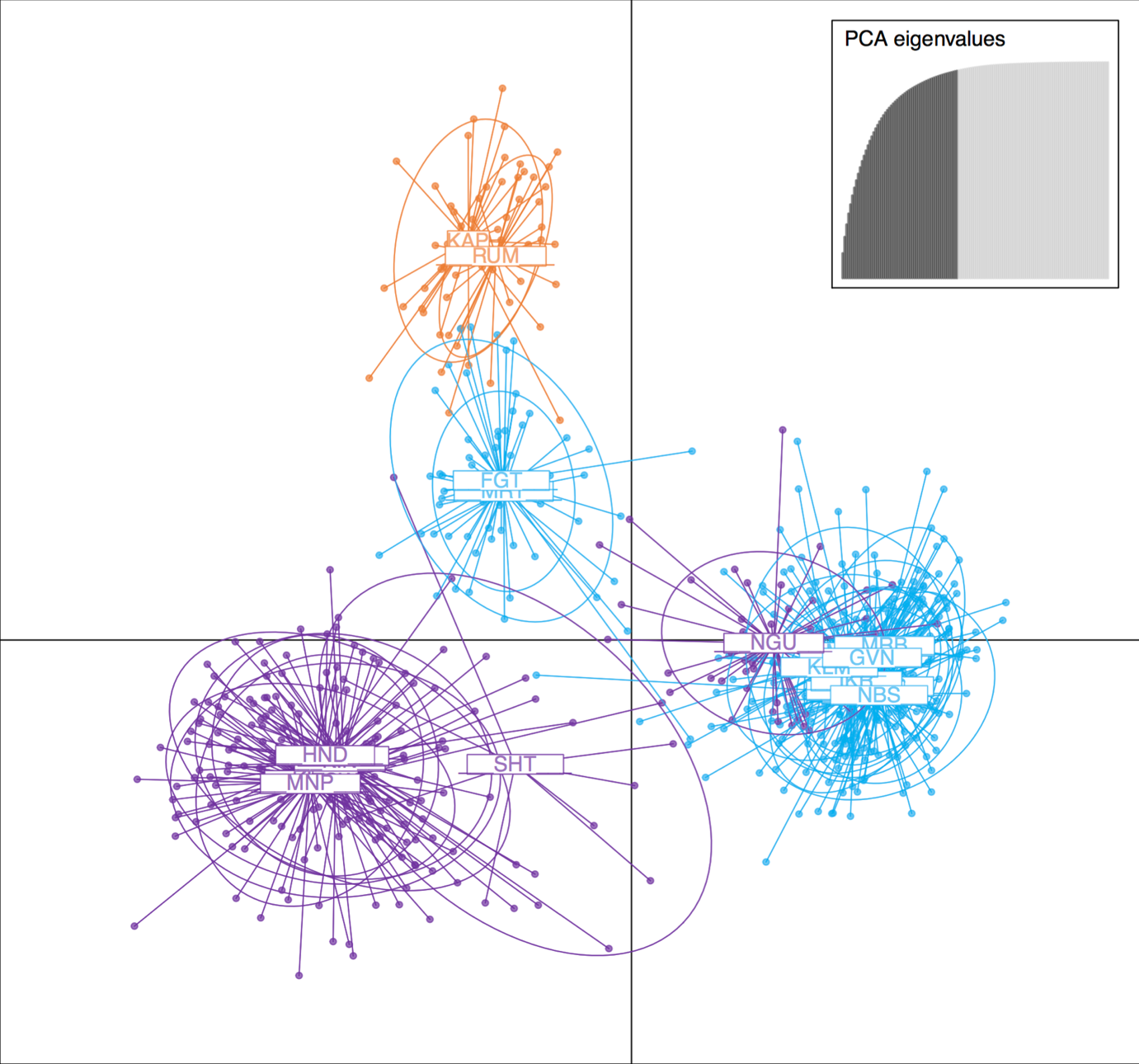


**S4 Fig.**

Supplement: S4 Fig — DAPC based on G. pallidipes microsatellite data for 21 sampling sites, completed in the R (R Development core team) using the “adegenet” package [63] with 40 principle components. Individuals are represented by dots linked by a line to the centroid and encompassed by 95% confidence intervals. Colors represent assignment to genetic cluster from the BAPS v 6 [55,56] analysis (orange = northwest, blue = southwest, purple = east). PCA eigenvalues represent variance explained by principle components, with the components included in the analysis shaded dark grey. (DOCX) [file pntd.0007855.s004.docx]

**
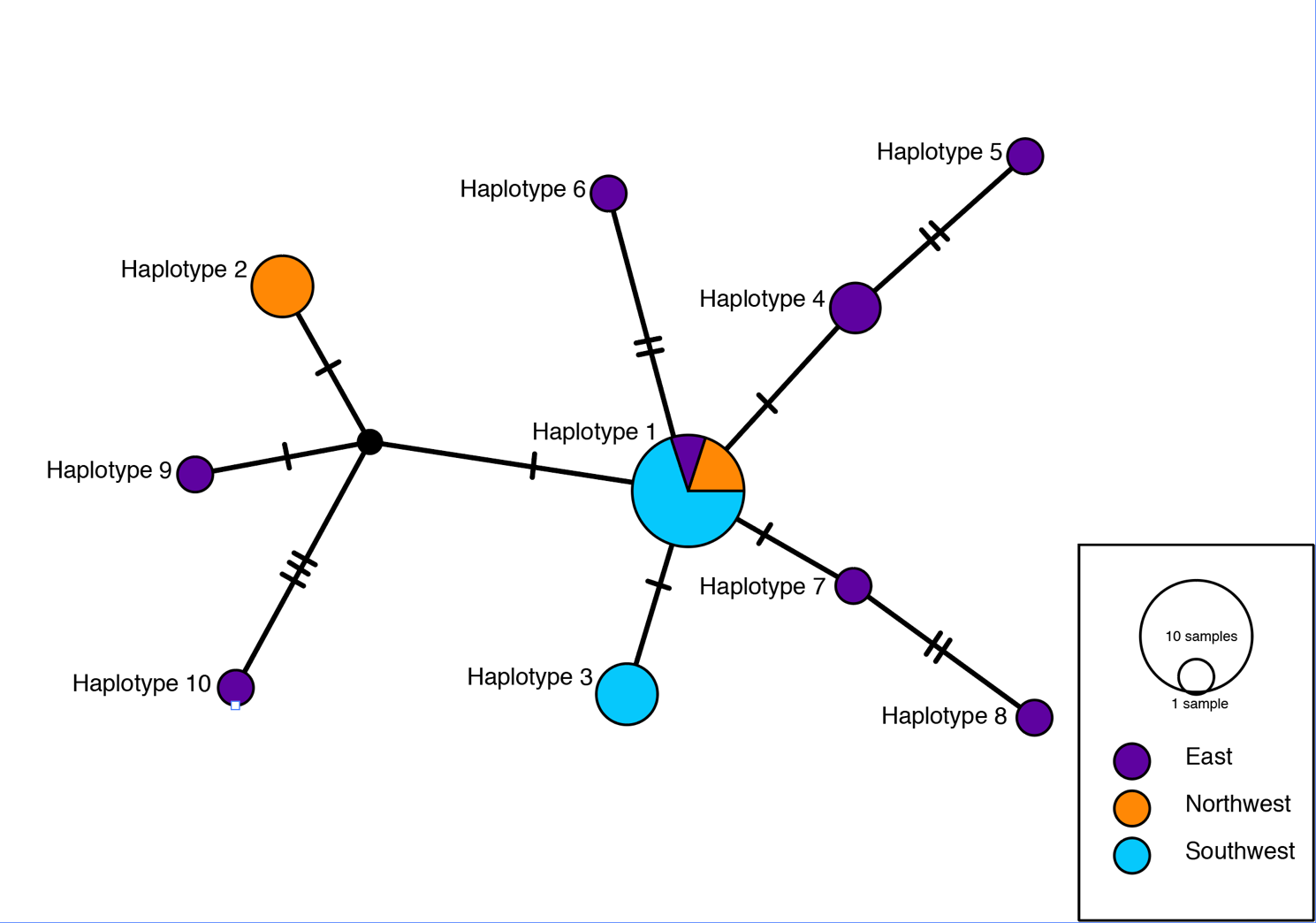
S5 Fig.**

Supplement: S5 Fig — TCS haplotype network where haplotypes are represented by circles that are sized proportionally to frequency and shaded with the genetic cluster they were chosen to represent in the ABC analysis. Hashes along the branches of the network represent a single nucleotide change (one inferred mutation), and black dots represent unsampled haplotypes. (DOCX) [file pntd.0007855.s005.docx]

**
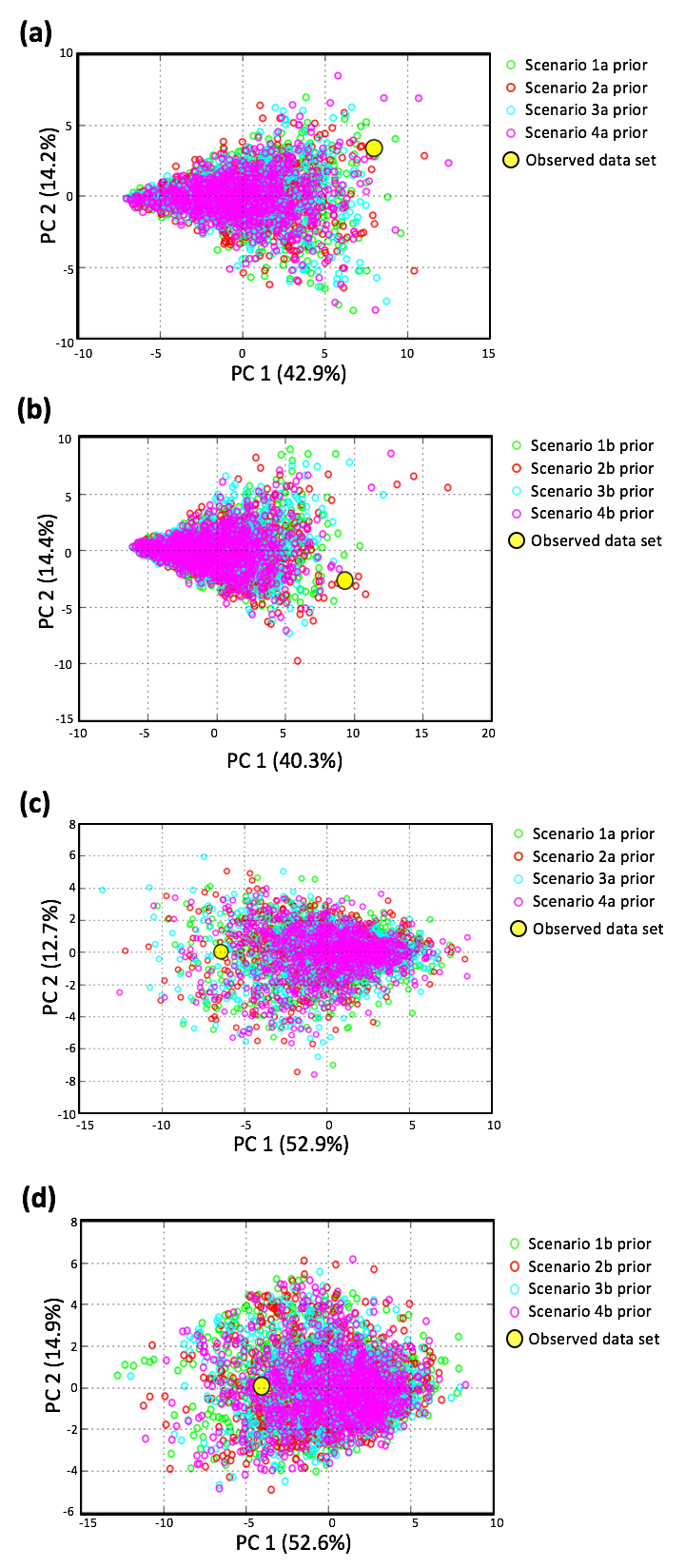
**

**S6 Fig.**

Supplement: S6 Fig — Principal components analysis (PCA) from (a) mtDNA under scenarios without fluctuating population sizes (Scenarios 1a, 2a, 3a, 4a), (b) mtDNA based results under scenarios with fluctuating population sizes (Scenarios 1b, 2b, 3b, 4b), (c) microsatellites based results under scenarios without fluctuating population sizes (Scenarios 1a, 2a, 3a, 4a), (b) microsatellite based results under scenarios with fluctuating population sizes (Scenarios 1b, 2b, 3b, 4b). Results from different scenarios are colored as indicated in the legend. ABC analyses was performed in DIYABC v2.0.4 [74]. (DOCX) [file pntd.0007855.s006.docx]

**
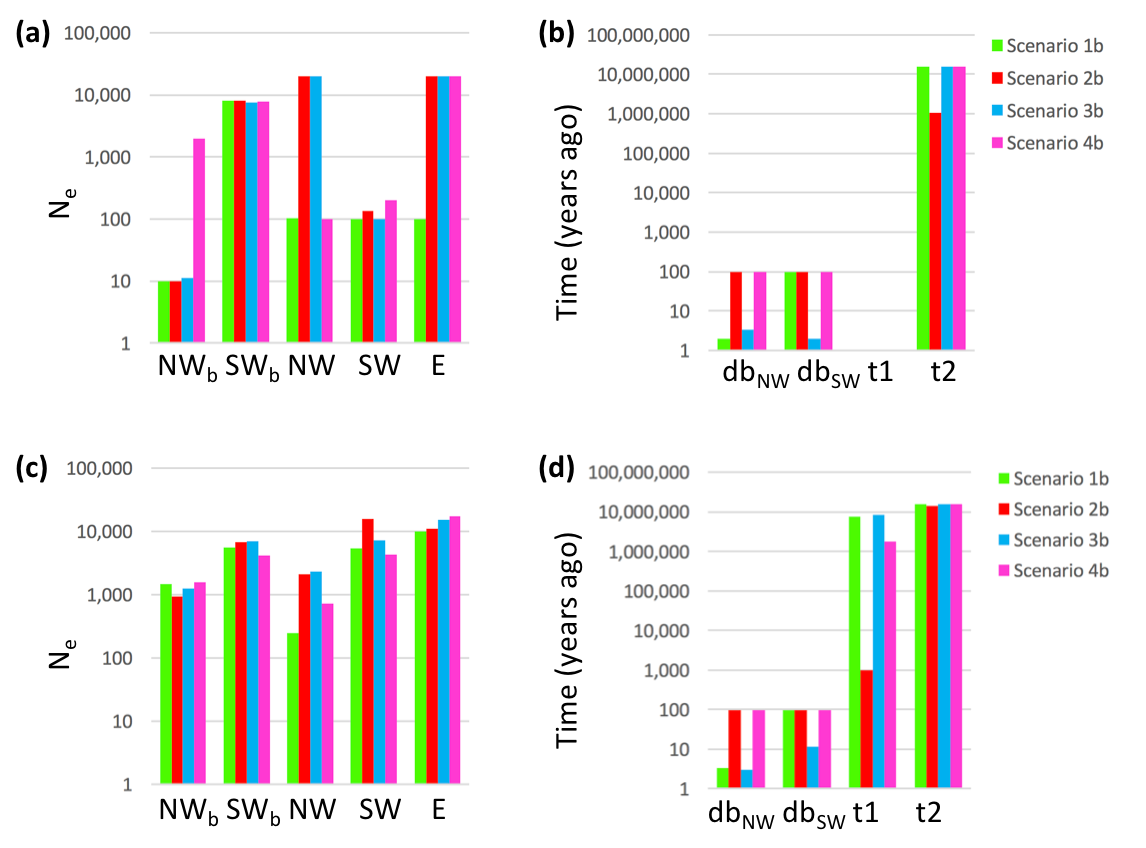
**

**S7 Fig.**

Supplement: S7 Fig — Mode of DIYABC v2.0.4 [74] parameter estimates from the winning scenarios of Analysis 2 (Scenarios 1b, 2b, 3b, 4b) in green, red, blue, and pink, respectively, including estimates of (a) population size from the mtDNA analysis (b) timing of simulated events from the mtDNA analysis, (c) population size from the microsatellite analysis, and (d) timing of simulated events from the microsatellite analysis plotted on a log scale to make all estimates visible in a single image. Population size estimates are presented for the northwest after a population bottleneck (NWb), the southwest after a population bottleneck (SWb), ancestral northwest (NW), ancestral southwest (SW), and the east (E). Timing estimates are presented for the date of bottleneck for the northwest (dbNW), date of bottleneck for the southwest (dbSW), the population split between the northwest and southwest (t1), and the population split between the west and east (t2). (DOCX) [file pntd.0007855.s007.docx]
