# Supplementary material for: Phylogeography and population structure of the tsetse fly Glossina pallidipes in Kenya and the Serengeti ecosystem
Source: PLoS Negl Trop Dis. 2020 Feb 24;14(2):e0007855. doi: 10.1371/journal.pntd.0007855 (PMC7058365; doi:10.1371/journal.pntd.0007855)
Supplement: S1 Table — Site name, site ID, county, latitude, longitude and sampling data of the 14 locations that did not have any flies during field collections despite past collection records that indicated the presence of G. pallidipes. (DOCX) [file pntd.0007855.s008.docx]

**S1 Table.**

| **Site** | **Site ID** | **County** | **Latitude** | **Longitude** | **Sampling date** |
| --- | --- | --- | --- | --- | --- |
| Rea Vipingo farm | RVF | Kilifi | -3.85002 | 39.781140 | Aug-15 |
| Kilifi plantations | KLP | Kilifi | -3.65986 | 39.864630 | Aug-15 |
| Arabuko Sokoke Forest | ASF | Kilifi | -3.26510 | 39.976440 | Aug-15 |
| Tsavo east | TSE | Taita Taveta | -3.16634 | 38.884210 | Aug-15 |
| Amboseli National Park | ANP | Kajiado | -2.70198 | 37.319550 | Oct-15 |
| Mwea National Reserve | MNR | Embu | -0.80577 | 37.592526 | Jan-16 |
| Luhano | LHM | Siaya | 0.016620 | 34.173033 | Jan-16 |
| Wire hills | WRH | Homabay | -0.46325 | 34.704400 | Jan-16 |
| Homa hills | HMH | Homabay | -0.37692 | 34.483750 | Jan-16 |
| Kabuto | KBT | Migori | -0.91367 | 34.164217 | Jan-16 |
| Kipangani | KPN | Kilifi | -2.79041 | 39.609550 | Jan-16 |
| Katotoi | KTT | Busia | 0.715370 | 34.314900 | Jan-16 |
| Adumai | ADM | Busia | 0.745830 | 34.313417 | Jan-16 |
| Kodera Forest | KDF | Homabay | -0.56608 | 34.674533 | Jan-16 |
