# Supplementary material for: Phylogeography and population structure of the tsetse fly Glossina pallidipes in Kenya and the Serengeti ecosystem
Source: PLoS Negl Trop Dis. 2020 Feb 24;14(2):e0007855. doi: 10.1371/journal.pntd.0007855 (PMC7058365; doi:10.1371/journal.pntd.0007855)
Supplement: S2 Table — The table shows loci names in the first column followed by fluorescent dye used for each locus, DNA sequences of the forward and reverse primers, size range of alleles in base pairs (bp), repeat motif length in base pairs and publication reference for primer design. (DOCX) [file pntd.0007855.s009.docx]

**S2 Table.**

| **Locus** | **Dye** | **Forward Primer** | **Reverse Primer** | **Frag.**  **length** | **Motif length** | **Repeat range** | **Source** |
| --- | --- | --- | --- | --- | --- | --- | --- |
| D05 | FAM | TTTCCTTCCAGACGAATCG | CTTGGTATGGTCGTACATGG | 94- 124 | 3 | 11 | Abila *et al.,* 2008 |
| GmmC17 | HEX | TGCGCTTTGAACGGAACG | CTATGCCGCCTGGCTTATC | 190-202 | 4 | 6 | Hyseni *et al*., 2011 |
| GmmK22 | TAM | ACGCTTACGTTTCCGTTACAC | AAGCTAACCGAACCAGCAC | 192-198 | 3 | 9 | Hyseni *et al.,* 2011 |
| GpCAG133 | HEX | ATTTTTGCGTCAACGTGA | GTTTATGAGGATGTTGTCCAGTTT | 185-209 | 3 | 11 | Baker *et al.,* 2001 |
| GmmL11 | HEX | CCACCACTAACAACGACAGC | TGGCTGGTTACAAGATTGCAC | 250-252 | 2 | 26 | Hyseni *et al*., 2011 |
| GmmA06 | TAM | ACTTCCATGTTATGTTCGTTGC | TGCCTTAGTTGAGAAACTCTGC | 154-166 | 2 | 17 | Hyseni  *et al.,* 2011 |
| GpC5b | NED | GTTGTTTTCTGCTCCTCAATA | GTTTCAAGGGTGTGTCGTCTTC | 187-239 | 3 | 9 | Ouma *et al.,* 2003 |
| GpA19a | FAM | CATATCCACACCCACATACAT | GTTTGCGATTATGGCTAGAGGTTT | 142-189 | 2 | 12 | Ouma *et al.,* 2003 |
| GpC10b | NED | GTTGATGTTGTGATGGTAATGA | GTTTGCTGGCAAAGAAACTAATGA | 283-314 | 3 | 7 | Ouma *et al.,* 2003 |
| GpB20b | FAM | AGTTTGCTTCTCAACGCAGTAG | GTTTCGGCAGTAGATGGCAA | 139-200 | 2 | 34 | Ouma *et al.,* 2003 |
| GpC26b | FAM | GGATCACCCTTCTTGAATG | GTTTGGACGTTATTTGTTCGTGTAA | 168-201 | 3 | 13 | Ouma *et al.,* 2003 |
