# Supplementary material for: Phylogeography and population structure of the tsetse fly Glossina pallidipes in Kenya and the Serengeti ecosystem
Source: PLoS Negl Trop Dis. 2020 Feb 24;14(2):e0007855. doi: 10.1371/journal.pntd.0007855 (PMC7058365; doi:10.1371/journal.pntd.0007855)
Supplement: S3 Table — ABC modeling was done using DIYABC v2.0.4 [74]. Panel (a) displays the prior minimum (min) and maximum (max) values of the priors used in the simulations and the scenarios these priors applied to. Panel (b) and (c) display results for the mtDNA and microsatellite based ABC analyses, respectively. Results displayed include the relative posterior probability of the scenario tested using the weighted logistic regression method described by [70], the lower 95% confidence interval of the posterior probability (CI), the upper CI, and the posterior predictive error (frequency of accepting a scenario other than the true scenario in 1000 runs of model checking with simulated data). All time priors (t1, t2) and the timing of the bottlenecks are displayed in years assuming a generation time of 5 per year. (DOCX) [file pntd.0007855.s010.docx]

**S3 Table**

| **(a)** |  |  |  | |  |
| --- | --- | --- | --- | --- | --- |
| **Parameter** | | | **Min** | **Max** | **Scenarios** |
| Ne northwest | | | 100 | 20,000 | All |
| Ne southwest | | | 100 | 20,000 | All |
| Ne east | | | 100 | 20,000 | All |
| t1 (northwest/southwest split) | | | 0 | 8,500,000 | All |
| t2 (west/east split) | | | 0 | 16,000,000 | All |
| Bottleneck Ne northwest* | | | 100 | 2,000 | 1b, 2b, 3b, 4b |
| Bottleneck Ne southwest* | | | 100 | 8,000 | 1b, 2b, 3b, 4b |
| Bottleneck timing northwest* | | | 0 | 100 | 1b, 2b, 3b, 4b |
| Bottleneck timing southwest* | | | 0 | 100 | 1b, 2b, 3b, 4b |
| mtDNA mutation rate | | | 1.00E-08 | 1.00E-05 | All |
| Microsatellite mutation rate | | | 1.00E-04 | 1.00E-03 | All |
| Microsatellite mutation step | | | 0.1 | 0.9 | All |
|  |  |  |  |  |  |
| **(b)** | **Scenario** | **Posterior probability** | **Lower CI** | **Upper CI** | **Posterior predictive error** |
| **Analysis 1** | **1a** | 0.2394 | 0.2199 | 0.2588 | 0.720 |
|  | **2a** | 0.2133 | 0.1965 | 0.2302 |  |
|  | **3a** | 0.2670 | 0.2479 | 0.2860 |  |
|  | **4a** | 0.2803 | 0.2607 | 0.3000 |  |
| **Analysis 2** | **1a** | 0.0002 | 0.0001 | 0.0003 | 0.209 |
|  | **1b** | 0.9998 | 0.9997 | 0.9999 |  |
|  | **2a** | 0.0002 | 0.0001 | 0.0002 | 0.222 |
|  | **2b** | 0.9998 | 0.9998 | 0.9999 |  |
|  | **3a** | 0.0001 | 0.0000 | 0.0002 | 0.206 |
|  | **3b** | 0.9999 | 0.9998 | 1.0000 |  |
|  | **4a** | 0.0002 | 0.0001 | 0.0003 | 0.213 |
|  | **4b** | 0.9998 | 0.9997 | 0.9999 |  |
|  |  |  |  |  |  |
| **(c)** | **Scenario** | **Posterior probability** | **Lower CI** | **Upper CI** | **Posterior predictive error** |
| **Analysis 1** | **1a** | 0.0695 | 0.0000 | 0.1467 | 0.749 |
|  | **2a** | 0.2920 | 0.1393 | 0.4447 |  |
|  | **3a** | 0.2070 | 0.0916 | 0.3225 |  |
|  | **4a** | 0.4315 | 0.2653 | 0.5976 |  |
| **Analysis 2** | **1a** | 0.0978 | 0.0811 | 0.1144 | 0.122 |
|  | **1b** | 0.9022 | 0.8856 | 0.9189 |  |
|  | **2a** | 0.5657 | 0.5072 | 0.6242 | 0.115 |
|  | **2b** | 0.4343 | 0.3758 | 0.4928 |  |
|  | **3a** | 0.7788 | 0.7225 | 0.8350 | 0.107 |
|  | **3b** | 0.2212 | 0.1650 | 0.2775 |  |
|  | **4a** | 0.3021 | 0.2681 | 0.3362 | 0.091 |
|  | **4b** | 0.6979 | 0.6638 | 0.7319 |  |
