# Supplementary material for: Phylogeography and population structure of the tsetse fly Glossina pallidipes in Kenya and the Serengeti ecosystem
Source: PLoS Negl Trop Dis. 2020 Feb 24;14(2):e0007855. doi: 10.1371/journal.pntd.0007855 (PMC7058365; doi:10.1371/journal.pntd.0007855)
Supplement: S4 Table — Bolded FIS values were considered significant after Benjamini-Hochberg correction for multiple testing. (DOCX) [file pntd.0007855.s011.docx]

**S4 Table**

|  | **Gmm C17** | **Gmm A06** | **Gmm L11** | **Gp C5b** | **Gmm K22** | **D05** | **Gp C26b** | **GpCAG 133** | **Gp A19a** | **Gp C10b** | **Gp B20b** |
| --- | --- | --- | --- | --- | --- | --- | --- | --- | --- | --- | --- |
|  |  |  |  |  |  |  |  |  |  |  |  |
| KAP | 0.216 | **0.271** | 0.112 | 0.132 | **0.111** | 0.230 | -0.102 | **0.586** | **0.612** | **0.240** | 0.048 |
| RUM | 0.360 | 0.026 | -0.018 | 0.151 | 0.067 | -0.074 | 0.120 | n/a | 0.327 | n/a | 0.091 |
| NGU | 0.185 | 0.078 | 0.047 | -0.069 | 0.129 | -0.004 | 0.138 | -0.033 | 0.115 | 0.116 | -0.040 |
| GVR | -0.018 | 0.132 | 0.297 | 0.228 | 0.141 | -0.015 | 0.271 | 0.140 | 0.560 | 0.026 | -0.002 |
| MRT | -0.018 | 0.044 | -0.049 | 0.194 | -0.107 | -0.025 | 0.356 | -0.162 | 0.044 | -0.010 | -0.076 |
| FGT | -0.018 | 0.018 | -0.002 | 0.042 | -0.229 | -0.057 | 0.283 | 0.164 | 0.481 | 0.003 | 0.112 |
| NBS | -0.018 | 0.169 | -0.029 | 0.196 | -0.023 | 0.066 | 0.335 | -0.097 | 0.433 | -0.187 | -0.023 |
| MRB | 0.517 | 0.222 | 0.215 | 0.112 | 0.081 | 0.081 | 0.199 | 0.191 | 0.347 | 0.138 | -0.053 |
| GTR | **0.373** | 0.044 | 0.067 | 0.228 | -0.338 | 0.036 | 0.004 | 0.232 | 0.178 | 0.259 | **0.146** |
| IKR | **0.478** | **0.270** | **0.218** | 0.064 | 0.079 | -0.085 | **0.278** | **0.227** | 0.254 | 0.128 | -0.163 |
| KLM | -0.094 | 0.231 | 0.120 | 0.010 | 0.277 | 0.006 | **0.305** | -0.003 | -0.085 | 0.022 | 0.024 |
| MSN | 0.230 | 0.019 | 0.043 | 0.042 | **0.445** | 0.252 | 0.102 | -0.041 | **0.301** | 0.081 | 0.105 |
| MSS | 0.659 | **0.389** | 0.193 | 0.115 | **-0.226** | -0.152 | 0.021 | 0.039 | 0.033 | -0.005 | 0.122 |
| NGK | n/a | **0.289** | 0.174 | 0.066 | -0.118 | -0.101 | **0.281** | 0.270 | 0.178 | -0.037 | -0.096 |
| MNP | -0.139 | 0.561 | -0.040 | 0.005 | 0.166 | -0.136 | 0.054 | -0.076 | 0.672 | -0.009 | 0.098 |
| KIB | 0.015 | 0.275 | 0.485 | -0.107 | 0.229 | -0.043 | -0.018 | -0.224 | 0.279 | 0.128 | 0.082 |
| TSW | -0.252 | 0.393 | 0.215 | 0.037 | 0.200 | -0.083 | 0.073 | -0.004 | 0.652 | 0.123 | -0.038 |
| KIN | -0.252 | 0.009 | 0.310 | 0.169 | 0.244 | -0.024 | 0.075 | -0.024 | 0.528 | 0.194 | 0.156 |
| SHT | n/a | 0.238 | 1.000 | 0.636 | 0.806 | 0.211 | 0.239 | 0.167 | 0.288 | 0.567 | 0.462 |
| SHI | 0.004 | -0.045 | 0.468 | 0.369 | 0.586 | 0.456 | -0.043 | 0.132 | 0.075 | 0.340 | 0.114 |
| HND | 0.049 | 0.407 | -0.034 | 0.127 | 0.299 | 0.478 | 0.052 | -0.140 | 0.488 | -0.187 | 0.166 |
