# Supplementary material for: Phylogeography and population structure of the tsetse fly Glossina pallidipes in Kenya and the Serengeti ecosystem
Source: PLoS Negl Trop Dis. 2020 Feb 24;14(2):e0007855. doi: 10.1371/journal.pntd.0007855 (PMC7058365; doi:10.1371/journal.pntd.0007855)
Supplement: S5 Table — (DOCX) [file pntd.0007855.s012.docx]

**S5 Table**

| **Locus pair** | **χ^2^** | **df** | **P-value** | **BH- P-value** |
| --- | --- | --- | --- | --- |
| D05 & GpC10b | 72.269 | 42 | 0.003 | 0.001 |
| GpC10b & GpC26b | 59.944 | 42 | 0.036 | 0.002 |
| GmmA06 & GpC5b | 56.707 | 42 | 0.064 | 0.003 |
| GpCAG133 & GmmA06 | 53.112 | 40 | 0.080 | 0.004 |
| D05 & GmmK22 | 55.136 | 42 | 0.084 | 0.005 |
| D05 & GmmC17 | 51.886 | 40 | 0.099 | 0.005 |
| GmmA06 & GpA19a | 53.880 | 42 | 0.103 | 0.006 |
| GmmC17 & GmmA06 | 51.447 | 40 | 0.106 | 0.007 |
| GmmA06 & GpC26b | 53.363 | 42 | 0.112 | 0.008 |
| D05 & GpCAG133 | 50.560 | 40 | 0.122 | 0.009 |
| D05 & GpA19a | 50.597 | 42 | 0.170 | 0.010 |
| GpCAG133 & GpA19a | 48.365 | 40 | 0.171 | 0.011 |
| GpCAG133 & GpC10b | 47.178 | 40 | 0.203 | 0.012 |
| GmmC17 & GpCAG133 | 44.524 | 38 | 0.216 | 0.013 |
| GmmC17 & GmmK22 | 46.152 | 40 | 0.233 | 0.014 |
| D05 & GpC5b | 47.236 | 42 | 0.267 | 0.015 |
| GmmA06 & GpC10b | 47.048 | 42 | 0.273 | 0.015 |
| GmmK22 & GpCAG133 | 44.533 | 40 | 0.287 | 0.016 |
| GmmC17 & GpA19a | 43.727 | 40 | 0.316 | 0.017 |
| GmmK22 & GpC26b | 44.637 | 42 | 0.362 | 0.018 |
| GmmC17 & GmmL11 | 41.952 | 40 | 0.386 | 0.019 |
| GmmC17 & GpC5b | 41.081 | 40 | 0.423 | 0.020 |
| GpCAG133 & GpC5b | 40.912 | 40 | 0.430 | 0.021 |
| GmmK22 & GpA19a | 42.901 | 42 | 0.432 | 0.022 |
| GpB20b & GpC26b | 42.522 | 42 | 0.449 | 0.023 |
| GmmL11 & GmmA06 | 40.367 | 40 | 0.454 | 0.024 |
| GpCAG133 & GmmL11 | 37.565 | 38 | 0.489 | 0.025 |
| GpC5b & GpC10b | 41.511 | 42 | 0.492 | 0.025 |
| GmmK22 & GpC5b | 40.488 | 42 | 0.537 | 0.026 |
| GmmK22 & GmmA06 | 39.505 | 42 | 0.581 | 0.027 |
| D05 & GmmA06 | 38.269 | 42 | 0.635 | 0.028 |
| GpC5b & GpB20b | 37.950 | 42 | 0.649 | 0.029 |
| GpA19a & GpC10b | 37.744 | 42 | 0.658 | 0.030 |
| GpC5b & GpC26b | 37.655 | 42 | 0.662 | 0.031 |
| GmmL11 & GpC10b | 35.285 | 40 | 0.682 | 0.032 |
| GmmL11 & GpC26b | 35.010 | 40 | 0.694 | 0.033 |
| D05 & GpC26b | 35.069 | 42 | 0.767 | 0.034 |
| GpCAG133 & GpB20b | 32.524 | 40 | 0.793 | 0.035 |
| GmmL11 & GpC5b | 32.398 | 40 | 0.798 | 0.035 |
| GmmK22 & GpC10b | 34.068 | 42 | 0.803 | 0.036 |
| GmmC17 & GpC10b | 32.205 | 40 | 0.805 | 0.037 |
| GmmC17 & GpB20b | 31.933 | 40 | 0.815 | 0.038 |
| GmmK22 & GpB20b | 32.034 | 42 | 0.867 | 0.039 |
| GmmK22 & GmmL11 | 29.514 | 40 | 0.888 | 0.040 |
| GpC5b & GpA19a | 30.670 | 42 | 0.902 | 0.041 |
| GpA19a & GpB20b | 30.540 | 42 | 0.905 | 0.042 |
| D05 & GmmL11 | 28.091 | 40 | 0.922 | 0.043 |
| GmmC17 & GpC26b | 27.357 | 40 | 0.936 | 0.044 |
| GmmL11 & GpB20b | 26.934 | 40 | 0.943 | 0.045 |
| GpCAG133 & GpC26b | 26.839 | 40 | 0.945 | 0.045 |
| GmmA06 & GpB20b | 28.192 | 42 | 0.949 | 0.046 |
| GpA19a & GpC26b | 28.106 | 42 | 0.951 | 0.047 |
| GpC10b & GpB20b | 27.826 | 42 | 0.955 | 0.048 |
| GmmL11 & GpA19a | 25.993 | 40 | 0.957 | 0.049 |
| D05 & GpB20b | 21.990 | 42 | 0.995 | 0.050 |
