# Supplementary material for: Phylogeography and population structure of the tsetse fly Glossina pallidipes in Kenya and the Serengeti ecosystem
Source: PLoS Negl Trop Dis. 2020 Feb 24;14(2):e0007855. doi: 10.1371/journal.pntd.0007855 (PMC7058365; doi:10.1371/journal.pntd.0007855)
Supplement: S6 Table — (DOCX) [file pntd.0007855.s013.docx]

**S6 Table**

| **Sampling site** | **Code** | **N** | **AR** | **H_O_** | **H_E_** | **F_IS_** | **F_IS_ p-value** |
| --- | --- | --- | --- | --- | --- | --- | --- |
| **Western cluster** |  |  |  |  |  |  |  |
| Kapesur | KAP | 8 | 2.29 | 0.44 | 0.60 | 0.28 | 0.00 |
| Ruma | RUM | 17 | 1.87 | 0.42 | 0.45 | 0.06 | 0.17 |
| Nguruman | NGU | 19 | 2.21 | 0.50 | 0.57 | 0.12 | 0.01 |
| ***Serengeti Ecosystem*** | |  |  |  |  |  |  |
| Governor’s Camp | GVR | 15 | 2.40 | 0.51 | 0.61 | 0.18 | 0.00 |
| Mara Talek | MRT | 9 | 2.36 | 0.56 | 0.60 | 0.07 | 0.16 |
| Fig Tree Camp | FGT | 16 | 2.43 | 0.52 | 0.61 | 0.16 | 0.00 |
| Naibosho | NBS | 14 | 2.35 | 0.54 | 0.65 | 0.18 | 0.00 |
| Marabridge | MRB | 13 | 2.59 | 0.48 | 0.67 | 0.29 | 0.00 |
| Grumeti | GTR | 11 | 2.57 | 0.53 | 0.70 | 0.22 | 0.00 |
| Ikorongo | IKR | 13 | 2.42 | 0.56 | 0.63 | 0.12 | 0.01 |
| Kilimafedha | KLM | 17 | 2.45 | 0.57 | 0.63 | 0.11 | 0.01 |
| Maswa North | MSN | 18 | 2.48 | 0.53 | 0.64 | 0.17 | 0.00 |
| Maswa South | MSS | 15 | 2.44 | 0.58 | 0.63 | 0.08 | 0.05 |
| Ngorongoro | NGK | 15 | 2.38 | 0.55 | 0.67 | 0.18 | 0.00 |
| **Eastern cluster** |  |  |  |  |  |  |  |
| Meru National Park | MNP | 9 | 2.55 | 0.54 | 0.65 | 0.19 | 0.00 |
| Kibwezi | KIB | 26 | 2.54 | 0.57 | 0.66 | 0.14 | 0.00 |
| Tsavo West | TSW | 27 | 2.55 | 0.57 | 0.66 | 0.14 | 0.00 |
| Kinango | KIN | 25 | 2.57 | 0.56 | 0.66 | 0.16 | 0.00 |
| Tiribe | SHT | 6 | 2.62 | 0.45 | 0.76 | 0.45 | 0.00 |
| Shimba | SHI | 20 | 2.54 | 0.52 | 0.67 | 0.22 | 0.00 |
| Hindi | HND | 17 | 2.54 | 0.59 | 0.66 | 0.11 | 0.01 |
