# Supplementary material for: Phylogeography and population structure of the tsetse fly Glossina pallidipes in Kenya and the Serengeti ecosystem
Source: PLoS Negl Trop Dis. 2020 Feb 24;14(2):e0007855. doi: 10.1371/journal.pntd.0007855 (PMC7058365; doi:10.1371/journal.pntd.0007855)
Supplement: S7 Table — Probability of assignment (q-value) for individuals to each of the four clusters identified in BAPS v 6 [55,56]. Admixed individuals (< 0.9 assignment probability to any one cluster) and those assigned to the outlier cluster are shown in bold. (DOCX) [file pntd.0007855.s014.docx]

**S7 Table.**

| **Sampling Site** | **Individual ID** | **q-values** | | | |
| --- | --- | --- | --- | --- | --- |
|  |  | **Northwest** | **Southwest** | **East** | **Outlier Cluster** |
| KAP | KAP001 | 1.00 | 0.00 | 0.00 | 0.00 |
| KAP | KAP002 | 1.00 | 0.00 | 0.00 | 0.00 |
| KAP | KAP003 | 1.00 | 0.00 | 0.00 | 0.00 |
| KAP | KAP004 | 1.00 | 0.00 | 0.00 | 0.00 |
| KAP | KAP005 | 1.00 | 0.00 | 0.00 | 0.00 |
| KAP | KAP006 | 1.00 | 0.00 | 0.00 | 0.00 |
| KAP | KAP007 | 1.00 | 0.00 | 0.00 | 0.00 |
| KAP | KAP008 | 1.00 | 0.00 | 0.00 | 0.00 |
| KAP | KAP009 | **0.00** | **0.00** | **0.00** | **1.00** |
| KAP | KAP010 | 1.00 | 0.00 | 0.00 | 0.00 |
| KAP | KAP011 | 1.00 | 0.00 | 0.00 | 0.00 |
| KAP | KAP012 | 1.00 | 0.00 | 0.00 | 0.00 |
| KAP | KAP013 | 1.00 | 0.00 | 0.00 | 0.00 |
| KAP | KAP014 | 1.00 | 0.00 | 0.00 | 0.00 |
| KAP | KAP015 | 1.00 | 0.00 | 0.00 | 0.00 |
| KAP | KAP051 | 1.00 | 0.00 | 0.00 | 0.00 |
| KAP | KAP052 | 1.00 | 0.00 | 0.00 | 0.00 |
| KAP | KAP053 | 1.00 | 0.00 | 0.00 | 0.00 |
| KAP | KAP054 | 1.00 | 0.00 | 0.00 | 0.00 |
| KAP | KAP055 | 1.00 | 0.00 | 0.00 | 0.00 |
| KAP | KAP056 | 1.00 | 0.00 | 0.00 | 0.00 |
| KAP | KAP057 | 1.00 | 0.00 | 0.00 | 0.00 |
| KAP | KAP058 | 1.00 | 0.00 | 0.00 | 0.00 |
| KAP | KAP059 | 1.00 | 0.00 | 0.00 | 0.00 |
| KAP | KAP060 | 1.00 | 0.00 | 0.00 | 0.00 |
| KAP | KAP061 | 1.00 | 0.00 | 0.00 | 0.00 |
| KAP | KAP062 | 1.00 | 0.00 | 0.00 | 0.00 |
| KAP | KAP064 | 1.00 | 0.00 | 0.00 | 0.00 |
| KAP | KAP065 | 1.00 | 0.00 | 0.00 | 0.00 |
| KAP | KAP066 | **0.00** | **0.00** | **0.00** | **1.00** |
| RUM | Ruma_006 | 1.00 | 0.00 | 0.00 | 0.00 |
| RUM | Ruma_007 | 1.00 | 0.00 | 0.00 | 0.00 |
| RUM | Ruma_008 | 1.00 | 0.00 | 0.00 | 0.00 |
| RUM | Ruma_009 | 1.00 | 0.00 | 0.00 | 0.00 |
| RUM | Ruma_010 | 1.00 | 0.00 | 0.00 | 0.00 |
| RUM | Ruma_011 | 1.00 | 0.00 | 0.00 | 0.00 |
| RUM | Ruma_012 | 1.00 | 0.00 | 0.00 | 0.00 |
| RUM | Ruma_013 | 1.00 | 0.00 | 0.00 | 0.00 |
| RUM | Ruma_014 | 1.00 | 0.00 | 0.00 | 0.00 |
| RUM | Ruma_017 | 1.00 | 0.00 | 0.00 | 0.00 |
| RUM | Ruma_018 | 1.00 | 0.00 | 0.00 | 0.00 |
| RUM | Ruma_019 | 1.00 | 0.00 | 0.00 | 0.00 |
| RUM | Ruma_001 | 1.00 | 0.00 | 0.00 | 0.00 |
| RUM | Ruma_002 | 1.00 | 0.00 | 0.00 | 0.00 |
| RUM | Ruma_003 | 1.00 | 0.00 | 0.00 | 0.00 |
| RUM | Ruma_004 | 1.00 | 0.00 | 0.00 | 0.00 |
| RUM | Ruma_005 | 1.00 | 0.00 | 0.00 | 0.00 |
| RUM | Ruma_015 | 1.00 | 0.00 | 0.00 | 0.00 |
| RUM | Ruma_016 | 1.00 | 0.00 | 0.00 | 0.00 |
| RUM | Ruma_028 | 1.00 | 0.00 | 0.00 | 0.00 |
| RUM | Ruma_032 | 1.00 | 0.00 | 0.00 | 0.00 |
| RUM | Ruma_035 | 1.00 | 0.00 | 0.00 | 0.00 |
| RUM | Ruma_039 | 1.00 | 0.00 | 0.00 | 0.00 |
| RUM | Ruma_040 | 1.00 | 0.00 | 0.00 | 0.00 |
| RUM | Ruma_021 | 1.00 | 0.00 | 0.00 | 0.00 |
| RUM | Ruma_022 | 1.00 | 0.00 | 0.00 | 0.00 |
| RUM | Ruma_024 | 1.00 | 0.00 | 0.00 | 0.00 |
| RUM | Ruma_025 | 1.00 | 0.00 | 0.00 | 0.00 |
| RUM | Ruma_027 | 1.00 | 0.00 | 0.00 | 0.00 |
| RUM | Ruma_029 | 1.00 | 0.00 | 0.00 | 0.00 |
| GVR | MMR142 | 0.00 | 1.00 | 0.00 | 0.00 |
| GVR | MMR147 | 0.00 | 1.00 | 0.00 | 0.00 |
| GVR | MMR148 | 0.00 | 1.00 | 0.00 | 0.00 |
| GVR | MMR150 | 0.00 | 1.00 | 0.00 | 0.00 |
| GVR | MMR151 | 0.00 | 1.00 | 0.00 | 0.00 |
| GVR | MMR153 | 0.00 | 1.00 | 0.00 | 0.00 |
| GVR | MMR154 | 0.00 | 1.00 | 0.00 | 0.00 |
| GVR | MMR155 | 0.00 | 1.00 | 0.00 | 0.00 |
| GVR | MMR157 | 0.00 | 1.00 | 0.00 | 0.00 |
| GVR | MMR159 | 0.00 | 1.00 | 0.00 | 0.00 |
| GVR | MMR162 | 0.00 | 1.00 | 0.00 | 0.00 |
| GVR | MMR164 | 0.00 | 1.00 | 0.00 | 0.00 |
| GVR | MMR165 | 0.00 | 1.00 | 0.00 | 0.00 |
| GVR | MMR166 | 0.00 | 1.00 | 0.00 | 0.00 |
| GVR | MMR167 | 0.00 | 1.00 | 0.00 | 0.00 |
| GVR | MMR176 | 0.00 | 1.00 | 0.00 | 0.00 |
| GVR | MMR177 | 0.00 | 1.00 | 0.00 | 0.00 |
| GVR | MMR178 | 0.00 | 1.00 | 0.00 | 0.00 |
| GVR | MMR181 | 0.00 | 1.00 | 0.00 | 0.00 |
| GVR | MMR183 | 0.00 | 1.00 | 0.00 | 0.00 |
| GVR | MMR184 | 0.00 | 1.00 | 0.00 | 0.00 |
| GVR | MMR186 | 0.00 | 1.00 | 0.00 | 0.00 |
| GVR | MMR187 | 0.00 | 1.00 | 0.00 | 0.00 |
| GVR | MMR188 | 0.00 | 1.00 | 0.00 | 0.00 |
| GVR | MMR191 | 0.00 | 1.00 | 0.00 | 0.00 |
| GVR | MMR192 | 0.00 | 1.00 | 0.00 | 0.00 |
| GVR | MMR194 | 0.00 | 1.00 | 0.00 | 0.00 |
| GVR | MMR196 | 0.00 | 1.00 | 0.00 | 0.00 |
| GVR | MMR197 | 0.00 | 1.00 | 0.00 | 0.00 |
| GVR | MMR198 | 0.00 | 1.00 | 0.00 | 0.00 |
| MRT | MMR071 | 0.00 | 1.00 | 0.00 | 0.00 |
| MRT | MMR072 | 0.00 | 1.00 | 0.00 | 0.00 |
| MRT | MMR074 | 0.00 | 1.00 | 0.00 | 0.00 |
| MRT | MMR075 | **0.54** | **0.42** | **0.04** | **0.00** |
| MRT | MMR076 | 0.00 | 1.00 | 0.00 | 0.00 |
| MRT | MMR077 | 0.00 | 1.00 | 0.00 | 0.00 |
| MRT | MMR078 | 0.00 | 1.00 | 0.00 | 0.00 |
| MRT | MMR079 | 0.00 | 1.00 | 0.00 | 0.00 |
| MRT | MMR081 | 0.00 | 1.00 | 0.00 | 0.00 |
| MRT | MMR082 | 0.00 | 1.00 | 0.00 | 0.00 |
| MRT | MMR083 | 0.00 | 1.00 | 0.00 | 0.00 |
| MRT | MMR084 | 0.00 | 1.00 | 0.00 | 0.00 |
| MRT | MMR085 | 0.00 | 1.00 | 0.00 | 0.00 |
| MRT | MMR086 | 0.00 | 1.00 | 0.00 | 0.00 |
| MRT | MMR087 | 0.00 | 1.00 | 0.00 | 0.00 |
| MRT | MMR109 | 0.00 | 1.00 | 0.00 | 0.00 |
| MRT | MMR110 | 0.00 | 1.00 | 0.00 | 0.00 |
| MRT | MMR111 | 0.00 | 1.00 | 0.00 | 0.00 |
| MRT | MMR113 | 0.00 | 1.00 | 0.00 | 0.00 |
| MRT | MMR114 | 0.00 | 1.00 | 0.00 | 0.00 |
| MRT | MMR115 | **0.42** | **0.58** | **0.00** | **0.00** |
| MRT | MMR116 | 0.00 | 1.00 | 0.00 | 0.00 |
| MRT | MMR117 | 0.00 | 1.00 | 0.00 | 0.00 |
| MRT | MMR118 | 0.00 | 1.00 | 0.00 | 0.00 |
| MRT | MMR119 | 0.00 | 1.00 | 0.00 | 0.00 |
| MRT | MMR121 | 0.00 | 1.00 | 0.00 | 0.00 |
| MRT | MMR122 | 0.00 | 1.00 | 0.00 | 0.00 |
| MRT | MMR124 | 0.00 | 1.00 | 0.00 | 0.00 |
| MRT | MMR125 | 0.00 | 1.00 | 0.00 | 0.00 |
| MRT | MMR127 | 0.00 | 1.00 | 0.00 | 0.00 |
| FGT | MMR001 | 0.00 | 1.00 | 0.00 | 0.00 |
| FGT | MMR002 | 0.00 | 1.00 | 0.00 | 0.00 |
| FGT | MMR004 | 0.00 | 1.00 | 0.00 | 0.00 |
| FGT | MMR005 | 0.00 | 1.00 | 0.00 | 0.00 |
| FGT | MMR006 | 0.00 | 1.00 | 0.00 | 0.00 |
| FGT | MMR007 | 0.00 | 1.00 | 0.00 | 0.00 |
| FGT | MMR009 | 0.00 | 1.00 | 0.00 | 0.00 |
| FGT | MMR010 | 0.00 | 1.00 | 0.00 | 0.00 |
| FGT | MMR011 | 0.00 | 1.00 | 0.00 | 0.00 |
| FGT | MMR012 | 0.00 | 1.00 | 0.00 | 0.00 |
| FGT | MMR013 | **0.60** | **0.39** | **0.01** | **0.00** |
| FGT | MMR014 | 0.00 | 1.00 | 0.00 | 0.00 |
| FGT | MMR015 | 0.00 | 1.00 | 0.00 | 0.00 |
| FGT | MMR016 | 0.94 | 0.02 | 0.04 | 0.00 |
| FGT | MMR017 | 0.00 | 1.00 | 0.00 | 0.00 |
| FGT | MMR018 | 0.00 | 1.00 | 0.00 | 0.00 |
| FGT | MMR020 | 0.00 | 1.00 | 0.00 | 0.00 |
| FGT | MMR021 | 0.00 | 1.00 | 0.00 | 0.00 |
| FGT | MMR022 | 0.00 | 1.00 | 0.00 | 0.00 |
| FGT | MMR023 | 0.00 | 1.00 | 0.00 | 0.00 |
| FGT | MMR036 | 0.00 | 1.00 | 0.00 | 0.00 |
| FGT | MMR037 | 0.00 | 1.00 | 0.00 | 0.00 |
| FGT | MMR042 | 0.00 | 1.00 | 0.00 | 0.00 |
| FGT | MMR043 | **0.17** | **0.30** | **0.53** | **0.00** |
| FGT | MMR044 | 0.00 | 1.00 | 0.00 | 0.00 |
| FGT | MMR053 | 0.00 | 1.00 | 0.00 | 0.00 |
| FGT | MMR048 | **0.53** | **0.44** | **0.03** | **0.00** |
| FGT | MMR049 | 0.00 | 1.00 | 0.00 | 0.00 |
| FGT | MMR050 | 0.00 | 1.00 | 0.00 | 0.00 |
| FGT | MMR051 | **0.52** | **0.43** | **0.05** | **0.00** |
| NBS | MMR283 | 0.00 | 1.00 | 0.00 | 0.00 |
| NBS | MMR284 | 0.00 | 1.00 | 0.00 | 0.00 |
| NBS | MMR287 | 0.00 | 1.00 | 0.00 | 0.00 |
| NBS | MMR289 | 0.00 | 1.00 | 0.00 | 0.00 |
| NBS | MMR290 | 0.00 | 1.00 | 0.00 | 0.00 |
| NBS | MMR291 | 0.00 | 1.00 | 0.00 | 0.00 |
| NBS | MMR293 | 0.00 | 1.00 | 0.00 | 0.00 |
| NBS | MMR294 | 0.00 | 1.00 | 0.00 | 0.00 |
| NBS | MMR295 | 0.00 | 1.00 | 0.00 | 0.00 |
| NBS | MMR296 | 0.00 | 1.00 | 0.00 | 0.00 |
| NBS | MMR297 | 0.00 | 1.00 | 0.00 | 0.00 |
| NBS | MMR298 | 0.00 | 1.00 | 0.00 | 0.00 |
| NBS | MMR299 | 0.00 | 1.00 | 0.00 | 0.00 |
| NBS | MMR300 | 0.00 | 1.00 | 0.00 | 0.00 |
| NBS | MMR301 | 0.00 | 1.00 | 0.00 | 0.00 |
| NBS | MMR319 | 0.00 | 1.00 | 0.00 | 0.00 |
| NBS | MMR320 | 0.00 | 1.00 | 0.00 | 0.00 |
| NBS | MMR322 | 0.00 | 1.00 | 0.00 | 0.00 |
| NBS | MMR323 | 0.00 | 1.00 | 0.00 | 0.00 |
| NBS | MMR324 | 0.00 | 1.00 | 0.00 | 0.00 |
| NBS | MMR326 | 0.00 | 1.00 | 0.00 | 0.00 |
| NBS | MMR327 | 0.00 | 1.00 | 0.00 | 0.00 |
| NBS | MMR328 | 0.00 | 1.00 | 0.00 | 0.00 |
| NBS | MMR332 | 0.00 | 1.00 | 0.00 | 0.00 |
| NBS | MMR333 | 0.00 | 1.00 | 0.00 | 0.00 |
| NBS | MMR334 | 0.00 | 1.00 | 0.00 | 0.00 |
| NBS | MMR335 | 0.00 | 1.00 | 0.00 | 0.00 |
| NBS | MMR341 | 0.00 | 1.00 | 0.00 | 0.00 |
| NBS | MMR342 | 0.00 | 1.00 | 0.00 | 0.00 |
| NBS | MMR343 | 0.00 | 1.00 | 0.00 | 0.00 |
| MRB | MMR211 | 0.00 | 1.00 | 0.00 | 0.00 |
| MRB | MMR212 | 0.00 | 1.00 | 0.00 | 0.00 |
| MRB | MMR214 | 0.00 | 1.00 | 0.00 | 0.00 |
| MRB | MMR215 | 0.00 | 1.00 | 0.00 | 0.00 |
| MRB | MMR216 | 0.00 | 1.00 | 0.00 | 0.00 |
| MRB | MMR217 | 0.00 | 1.00 | 0.00 | 0.00 |
| MRB | MMR220 | 0.00 | 1.00 | 0.00 | 0.00 |
| MRB | MMR222 | 0.00 | 1.00 | 0.00 | 0.00 |
| MRB | MMR227 | 0.00 | 1.00 | 0.00 | 0.00 |
| MRB | MMR228 | 0.00 | 1.00 | 0.00 | 0.00 |
| MRB | MMR229 | 0.00 | 1.00 | 0.00 | 0.00 |
| MRB | MMR231 | 0.00 | 1.00 | 0.00 | 0.00 |
| MRB | MMR233 | 0.00 | 1.00 | 0.00 | 0.00 |
| MRB | MMR234 | 0.00 | 1.00 | 0.00 | 0.00 |
| MRB | MMR235 | 0.00 | 1.00 | 0.00 | 0.00 |
| MRB | MMR246 | 0.00 | 1.00 | 0.00 | 0.00 |
| MRB | MMR249 | 0.00 | 1.00 | 0.00 | 0.00 |
| MRB | MMR250 | 0.00 | 1.00 | 0.00 | 0.00 |
| MRB | MMR252 | 0.00 | 1.00 | 0.00 | 0.00 |
| MRB | MMR255 | 0.00 | 1.00 | 0.00 | 0.00 |
| MRB | MMR259 | 0.00 | 1.00 | 0.00 | 0.00 |
| MRB | MMR260 | 0.00 | 1.00 | 0.00 | 0.00 |
| MRB | MMR261 | 0.00 | 1.00 | 0.00 | 0.00 |
| MRB | MMR263 | 0.00 | 1.00 | 0.00 | 0.00 |
| MRB | MMR265 | 0.00 | 1.00 | 0.00 | 0.00 |
| MRB | MMR266 | 0.00 | 1.00 | 0.00 | 0.00 |
| MRB | MMR267 | 0.00 | 1.00 | 0.00 | 0.00 |
| MRB | MMR271 | 0.00 | 1.00 | 0.00 | 0.00 |
| MRB | MMR272 | 0.00 | 1.00 | 0.00 | 0.00 |
| MRB | MMR273 | 0.00 | 1.00 | 0.00 | 0.00 |
| GTR | GTR001 | 0.00 | 1.00 | 0.00 | 0.00 |
| GTR | GTR002 | 0.00 | 1.00 | 0.00 | 0.00 |
| GTR | GTR003 | 0.00 | 1.00 | 0.00 | 0.00 |
| GTR | GTR004 | 0.00 | 1.00 | 0.00 | 0.00 |
| GTR | GTR005 | 0.00 | 1.00 | 0.00 | 0.00 |
| GTR | GTR006 | 0.00 | 1.00 | 0.00 | 0.00 |
| GTR | GTR007 | 0.00 | 1.00 | 0.00 | 0.00 |
| GTR | GTR008 | 0.00 | 1.00 | 0.00 | 0.00 |
| GTR | GTR009 | 0.00 | 1.00 | 0.00 | 0.00 |
| GTR | GTR010 | 0.00 | 1.00 | 0.00 | 0.00 |
| GTR | GTR011 | 0.00 | 1.00 | 0.00 | 0.00 |
| GTR | GTR012 | 0.00 | 1.00 | 0.00 | 0.00 |
| GTR | GTR013 | 0.00 | 1.00 | 0.00 | 0.00 |
| GTR | GTR014 | 0.00 | 1.00 | 0.00 | 0.00 |
| GTR | GTR015 | 0.00 | 1.00 | 0.00 | 0.00 |
| GTR | GTR021 | 0.00 | 1.00 | 0.00 | 0.00 |
| GTR | GTR022 | 0.00 | 1.00 | 0.00 | 0.00 |
| GTR | GTR023 | 0.00 | 1.00 | 0.00 | 0.00 |
| GTR | GTR024 | 0.00 | 1.00 | 0.00 | 0.00 |
| GTR | GTR025 | 0.00 | 1.00 | 0.00 | 0.00 |
| GTR | GTR026 | 0.00 | 1.00 | 0.00 | 0.00 |
| GTR | GTR027 | 0.00 | 1.00 | 0.00 | 0.00 |
| GTR | GTR028 | 0.00 | 1.00 | 0.00 | 0.00 |
| GTR | GTR029 | 0.00 | 1.00 | 0.00 | 0.00 |
| GTR | GTR030 | 0.00 | 1.00 | 0.00 | 0.00 |
| GTR | GTR031 | 0.00 | 1.00 | 0.00 | 0.00 |
| GTR | GTR032 | 0.00 | 1.00 | 0.00 | 0.00 |
| GTR | GTR033 | 0.00 | 1.00 | 0.00 | 0.00 |
| GTR | GTR034 | 0.00 | 1.00 | 0.00 | 0.00 |
| GTR | GTR035 | 0.00 | 1.00 | 0.00 | 0.00 |
| IKR | IKR001 | 0.00 | 1.00 | 0.00 | 0.00 |
| IKR | IKR002 | **0.00** | **0.00** | **0.00** | **1.00** |
| IKR | IKR003 | 0.00 | 1.00 | 0.00 | 0.00 |
| IKR | IKR004 | 0.00 | 1.00 | 0.00 | 0.00 |
| IKR | IKR005 | 0.00 | 1.00 | 0.00 | 0.00 |
| IKR | IKR006 | 0.00 | 1.00 | 0.00 | 0.00 |
| IKR | IKR007 | 0.00 | 1.00 | 0.00 | 0.00 |
| IKR | IKR008 | 0.00 | 1.00 | 0.00 | 0.00 |
| IKR | IKR009 | 0.00 | 1.00 | 0.00 | 0.00 |
| IKR | IKR010 | 0.00 | 1.00 | 0.00 | 0.00 |
| IKR | IKR011 | 0.00 | 1.00 | 0.00 | 0.00 |
| IKR | IKR012 | 0.00 | 1.00 | 0.00 | 0.00 |
| IKR | IKR013 | **0.00** | **0.00** | **0.00** | **1.00** |
| IKR | IKR015 | 0.00 | 1.00 | 0.00 | 0.00 |
| IKR | IKR016 | 0.00 | 1.00 | 0.00 | 0.00 |
| IKR | IKR017 | 0.00 | 1.00 | 0.00 | 0.00 |
| IKR | IKR018 | 0.00 | 1.00 | 0.00 | 0.00 |
| IKR | IKR019 | 0.00 | 1.00 | 0.00 | 0.00 |
| IKR | IKR020 | 0.00 | 1.00 | 0.00 | 0.00 |
| IKR | IKR021 | 0.00 | 1.00 | 0.00 | 0.00 |
| IKR | IKR022 | 0.00 | 1.00 | 0.00 | 0.00 |
| IKR | IKR023 | 0.00 | 1.00 | 0.00 | 0.00 |
| IKR | IKR024 | 0.00 | 1.00 | 0.00 | 0.00 |
| IKR | IKR025 | 0.00 | 1.00 | 0.00 | 0.00 |
| IKR | IKR026 | 0.00 | 1.00 | 0.00 | 0.00 |
| IKR | IKR027 | 0.00 | 1.00 | 0.00 | 0.00 |
| IKR | IKR028 | 0.00 | 1.00 | 0.00 | 0.00 |
| IKR | IKR029 | 0.00 | 1.00 | 0.00 | 0.00 |
| IKR | IKR030 | 0.00 | 1.00 | 0.00 | 0.00 |
| IKR | IKR031 | 0.00 | 1.00 | 0.00 | 0.00 |
| KLM | SEKF001 | 0.00 | 1.00 | 0.00 | 0.00 |
| KLM | SEKF002 | **0.10** | **0.31** | **0.59** | **0.00** |
| KLM | SEKF003 | 0.00 | 1.00 | 0.00 | 0.00 |
| KLM | SEKF004 | 0.00 | 1.00 | 0.00 | 0.00 |
| KLM | SEKF005 | 0.00 | 1.00 | 0.00 | 0.00 |
| KLM | SEKF006 | 0.00 | 1.00 | 0.00 | 0.00 |
| KLM | SEKF007 | 0.00 | 1.00 | 0.00 | 0.00 |
| KLM | SEKF008 | 0.00 | 1.00 | 0.00 | 0.00 |
| KLM | SEKF009 | 0.00 | 1.00 | 0.00 | 0.00 |
| KLM | SEKF010 | 0.00 | 1.00 | 0.00 | 0.00 |
| KLM | SEKF011 | 0.00 | 1.00 | 0.00 | 0.00 |
| KLM | SEKF012 | 0.00 | 1.00 | 0.00 | 0.00 |
| KLM | SEKF013 | 0.00 | 1.00 | 0.00 | 0.00 |
| KLM | SEKF014 | 0.00 | 1.00 | 0.00 | 0.00 |
| KLM | SEKF015 | 0.00 | 1.00 | 0.00 | 0.00 |
| KLM | SEKF016 | 0.00 | 1.00 | 0.00 | 0.00 |
| KLM | SEKF017 | 0.00 | 1.00 | 0.00 | 0.00 |
| KLM | SEKF018 | 0.00 | 1.00 | 0.00 | 0.00 |
| KLM | SEKF019 | 0.00 | 1.00 | 0.00 | 0.00 |
| KLM | SEKF020 | 0.00 | 1.00 | 0.00 | 0.00 |
| KLM | SEKF021 | 0.00 | 1.00 | 0.00 | 0.00 |
| KLM | SEKF022 | 0.00 | 1.00 | 0.00 | 0.00 |
| KLM | SEKF023 | 0.00 | 1.00 | 0.00 | 0.00 |
| KLM | SEKF024 | 0.00 | 1.00 | 0.00 | 0.00 |
| KLM | SEKF025 | 0.00 | 1.00 | 0.00 | 0.00 |
| KLM | SEKF026 | 0.00 | 1.00 | 0.00 | 0.00 |
| KLM | SEKF027 | 0.00 | 1.00 | 0.00 | 0.00 |
| KLM | SEKF028 | 0.00 | 1.00 | 0.00 | 0.00 |
| KLM | SEKF029 | 0.00 | 1.00 | 0.00 | 0.00 |
| KLM | SEKF030 | 0.00 | 1.00 | 0.00 | 0.00 |
| MSN | MSA001 | 0.00 | 1.00 | 0.00 | 0.00 |
| MSN | MSA002 | 0.00 | 1.00 | 0.00 | 0.00 |
| MSN | MSA003 | 0.00 | 1.00 | 0.00 | 0.00 |
| MSN | MSA004 | 0.00 | 1.00 | 0.00 | 0.00 |
| MSN | MSA005 | 0.00 | 1.00 | 0.00 | 0.00 |
| MSN | MSA006 | 0.00 | 1.00 | 0.00 | 0.00 |
| MSN | MSA007 | 0.00 | 1.00 | 0.00 | 0.00 |
| MSN | MSA008 | 0.00 | 1.00 | 0.00 | 0.00 |
| MSN | MSA009 | 0.00 | 1.00 | 0.00 | 0.00 |
| MSN | MSA010 | 0.00 | 1.00 | 0.00 | 0.00 |
| MSN | MSA011 | 0.00 | 1.00 | 0.00 | 0.00 |
| MSN | MSA012 | 0.00 | 1.00 | 0.00 | 0.00 |
| MSN | MSA013 | 0.00 | 1.00 | 0.00 | 0.00 |
| MSN | MSA014 | 0.00 | 1.00 | 0.00 | 0.00 |
| MSN | MSA015 | 0.00 | 1.00 | 0.00 | 0.00 |
| MSN | MSA024 | 0.00 | 1.00 | 0.00 | 0.00 |
| MSN | MSA025 | 0.00 | 1.00 | 0.00 | 0.00 |
| MSN | MSA026 | 0.00 | 1.00 | 0.00 | 0.00 |
| MSN | MSA027 | 0.00 | 1.00 | 0.00 | 0.00 |
| MSN | MSA028 | 0.00 | 1.00 | 0.00 | 0.00 |
| MSN | MSA029 | 0.00 | 1.00 | 0.00 | 0.00 |
| MSN | MSA030 | 0.00 | 1.00 | 0.00 | 0.00 |
| MSN | MSA031 | 0.00 | 1.00 | 0.00 | 0.00 |
| MSN | MSA032 | 0.00 | 1.00 | 0.00 | 0.00 |
| MSN | MSA033 | 0.00 | 1.00 | 0.00 | 0.00 |
| MSN | MSA034 | 0.00 | 1.00 | 0.00 | 0.00 |
| MSN | MSA035 | 0.00 | 1.00 | 0.00 | 0.00 |
| MSN | MSA036 | 0.00 | 1.00 | 0.00 | 0.00 |
| MSN | MSA037 | 0.00 | 1.00 | 0.00 | 0.00 |
| MSN | MSA038 | 0.00 | 1.00 | 0.00 | 0.00 |
| MSS | MSB001 | 0.00 | 1.00 | 0.00 | 0.00 |
| MSS | MSB002 | 0.00 | 1.00 | 0.00 | 0.00 |
| MSS | MSB003 | 0.00 | 1.00 | 0.00 | 0.00 |
| MSS | MSB004 | 0.00 | 1.00 | 0.00 | 0.00 |
| MSS | MSB005 | 0.00 | 1.00 | 0.00 | 0.00 |
| MSS | MSB006 | 0.00 | 1.00 | 0.00 | 0.00 |
| MSS | MSB007 | 0.00 | 1.00 | 0.00 | 0.00 |
| MSS | MSB008 | 0.00 | 1.00 | 0.00 | 0.00 |
| MSS | MSB009 | 0.00 | 1.00 | 0.00 | 0.00 |
| MSS | MSB010 | 0.00 | 1.00 | 0.00 | 0.00 |
| MSS | MSB011 | 0.00 | 1.00 | 0.00 | 0.00 |
| MSS | MSB012 | 0.00 | 1.00 | 0.00 | 0.00 |
| MSS | MSB013 | 0.00 | 1.00 | 0.00 | 0.00 |
| MSS | MSB014 | 0.00 | 1.00 | 0.00 | 0.00 |
| MSS | MSB015 | 0.00 | 1.00 | 0.00 | 0.00 |
| MSS | MSB051 | 0.00 | 1.00 | 0.00 | 0.00 |
| MSS | MSB052 | 0.00 | 1.00 | 0.00 | 0.00 |
| MSS | MSB053 | 0.00 | 1.00 | 0.00 | 0.00 |
| MSS | MSB054 | 0.00 | 1.00 | 0.00 | 0.00 |
| MSS | MSB055 | 0.00 | 1.00 | 0.00 | 0.00 |
| MSS | MSB056 | 0.00 | 1.00 | 0.00 | 0.00 |
| MSS | MSB057 | 0.00 | 1.00 | 0.00 | 0.00 |
| MSS | MSB058 | 0.00 | 1.00 | 0.00 | 0.00 |
| MSS | MSB059 | 0.00 | 1.00 | 0.00 | 0.00 |
| MSS | MSB060 | 0.00 | 1.00 | 0.00 | 0.00 |
| MSS | MSB061 | **0.18** | **0.34** | **0.48** | **0.00** |
| MSS | MSB062 | 0.00 | 1.00 | 0.00 | 0.00 |
| MSS | MSB063 | 0.00 | 1.00 | 0.00 | 0.00 |
| MSS | MSB064 | 0.00 | 1.00 | 0.00 | 0.00 |
| MSS | MSB065 | 0.00 | 1.00 | 0.00 | 0.00 |
| NGK | NGK001 | 0.00 | 1.00 | 0.00 | 0.00 |
| NGK | NGK002 | 0.00 | 1.00 | 0.00 | 0.00 |
| NGK | NGK003 | 0.00 | 1.00 | 0.00 | 0.00 |
| NGK | NGK004 | 0.00 | 1.00 | 0.00 | 0.00 |
| NGK | NGK005 | 0.00 | 1.00 | 0.00 | 0.00 |
| NGK | NGK006 | 0.00 | 1.00 | 0.00 | 0.00 |
| NGK | NGK007 | 0.00 | 1.00 | 0.00 | 0.00 |
| NGK | NGK008 | 0.00 | 1.00 | 0.00 | 0.00 |
| NGK | NGK009 | 0.04 | 0.00 | 0.96 | 0.00 |
| NGK | NGK010 | 0.00 | 1.00 | 0.00 | 0.00 |
| NGK | NGK011 | 0.00 | 1.00 | 0.00 | 0.00 |
| NGK | NGK013 | 0.00 | 1.00 | 0.00 | 0.00 |
| NGK | NGK014 | 0.00 | 1.00 | 0.00 | 0.00 |
| NGK | NGK015 | 0.00 | 1.00 | 0.00 | 0.00 |
| NGK | NGK016 | 0.00 | 1.00 | 0.00 | 0.00 |
| NGK | NGK025 | 0.00 | 1.00 | 0.00 | 0.00 |
| NGK | NGK026 | 0.00 | 1.00 | 0.00 | 0.00 |
| NGK | NGK027 | 0.00 | 1.00 | 0.00 | 0.00 |
| NGK | NGK028 | 0.00 | 1.00 | 0.00 | 0.00 |
| NGK | NGK029 | 0.00 | 1.00 | 0.00 | 0.00 |
| NGK | NGK030 | 0.00 | 1.00 | 0.00 | 0.00 |
| NGK | NGK031 | 0.00 | 1.00 | 0.00 | 0.00 |
| NGK | NGK032 | 0.00 | 1.00 | 0.00 | 0.00 |
| NGK | NGK033 | 0.00 | 1.00 | 0.00 | 0.00 |
| NGK | NGK034 | 0.00 | 1.00 | 0.00 | 0.00 |
| NGK | NGK035 | 0.00 | 1.00 | 0.00 | 0.00 |
| NGK | NGK036 | 0.00 | 1.00 | 0.00 | 0.00 |
| NGK | NGK037 | 0.00 | 1.00 | 0.00 | 0.00 |
| NGK | NGK038 | 0.00 | 1.00 | 0.00 | 0.00 |
| NGK | NGK039 | 0.00 | 1.00 | 0.00 | 0.00 |
| NGU | MOK10 | 0.00 | 0.00 | 1.00 | 0.00 |
| NGU | MOK11 | 0.00 | 0.00 | 1.00 | 0.00 |
| NGU | MOK12 | 0.00 | 0.00 | 1.00 | 0.00 |
| NGU | MOK13 | 0.00 | 0.00 | 1.00 | 0.00 |
| NGU | MOK14 | 0.00 | 0.00 | 1.00 | 0.00 |
| NGU | MOK15 | 0.00 | 0.00 | 1.00 | 0.00 |
| NGU | MOK16 | **0.00** | **0.86** | **0.14** | **0.00** |
| NGU | MOK17 | 0.00 | 0.00 | 1.00 | 0.00 |
| NGU | MOK19 | 0.00 | 0.00 | 1.00 | 0.00 |
| NGU | MOK20 | 0.00 | 0.92 | 0.08 | 0.00 |
| NGU | MOK21 | 0.00 | 0.00 | 1.00 | 0.00 |
| NGU | MOK22 | 0.00 | 0.00 | 1.00 | 0.00 |
| NGU | MOK23 | 0.00 | 0.00 | 1.00 | 0.00 |
| NGU | MOK24 | 0.00 | 0.00 | 1.00 | 0.00 |
| NGU | MOK25 | 0.00 | 0.00 | 1.00 | 0.00 |
| NGU | MOK26 | 0.00 | 0.00 | 1.00 | 0.00 |
| NGU | MOK27 | 0.00 | 0.00 | 1.00 | 0.00 |
| NGU | MOK28 | 0.00 | 0.00 | 1.00 | 0.00 |
| NGU | MOK29 | 0.00 | 0.00 | 1.00 | 0.00 |
| NGU | MOK30 | 0.00 | 0.00 | 1.00 | 0.00 |
| NGU | MOK31 | 0.00 | 0.00 | 1.00 | 0.00 |
| NGU | MOK32 | **0.48** | **0.18** | **0.34** | **0.00** |
| NGU | MOK33 | 0.00 | 0.00 | 1.00 | 0.00 |
| NGU | MOK34 | 0.00 | 0.00 | 1.00 | 0.00 |
| NGU | MOK35 | 0.00 | 0.00 | 1.00 | 0.00 |
| NGU | MOK36 | 0.00 | 0.00 | 1.00 | 0.00 |
| NGU | MOK37 | 0.00 | 0.00 | 1.00 | 0.00 |
| NGU | MOK38 | 0.00 | 0.00 | 1.00 | 0.00 |
| NGU | MOK39 | 0.00 | 0.00 | 1.00 | 0.00 |
| NGU | MOK40 | 0.00 | 0.00 | 1.00 | 0.00 |
| NGU | MOK41 | 0.00 | 0.00 | 1.00 | 0.00 |
| NGU | MOK42 | **0.00** | **0.83** | **0.17** | **0.00** |
| NGU | MOK43 | **0.44** | **0.02** | **0.54** | **0.00** |
| NGU | MOK44 | 0.00 | 0.00 | 1.00 | 0.00 |
| NGU | MOK45 | 0.00 | 0.00 | 1.00 | 0.00 |
| NGU | MOK46 | 0.00 | 0.00 | 1.00 | 0.00 |
| NGU | MOK47 | **0.15** | **0.69** | **0.16** | **0.00** |
| NGU | MOK48 | 0.00 | 0.00 | 1.00 | 0.00 |
| NGU | MOK49 | 0.00 | 0.00 | 1.00 | 0.00 |
| NGU | MOK50 | 0.00 | 0.00 | 1.00 | 0.00 |
| NGU | MOK51 | 0.00 | 0.00 | 1.00 | 0.00 |
| NGU | MOK52 | **0.20** | **0.60** | **0.20** | **0.00** |
| NGU | MOK53 | 0.00 | 0.00 | 1.00 | 0.00 |
| NGU | MOK54 | 0.00 | 0.00 | 1.00 | 0.00 |
| NGU | MOK55 | 0.00 | 0.00 | 1.00 | 0.00 |
| NGU | MOK6 | **0.34** | **0.49** | **0.17** | **0.00** |
| NGU | MOK7 | 0.00 | 0.00 | 1.00 | 0.00 |
| NGU | MOK8 | 0.00 | 0.00 | 1.00 | 0.00 |
| MNP | MNP_002 | 0.00 | 0.00 | 1.00 | 0.00 |
| MNP | MNP_003 | 0.00 | 0.00 | 1.00 | 0.00 |
| MNP | MNP_005 | 0.00 | 0.00 | 1.00 | 0.00 |
| MNP | MNP_006 | 0.00 | 0.00 | 1.00 | 0.00 |
| MNP | MNP_008 | 0.00 | 0.00 | 1.00 | 0.00 |
| MNP | MNP_012 | 0.00 | 0.00 | 1.00 | 0.00 |
| MNP | MNP_013 | 0.00 | 0.00 | 1.00 | 0.00 |
| MNP | MNP_014 | 0.00 | 0.00 | 1.00 | 0.00 |
| MNP | MNP_015 | 0.00 | 0.00 | 1.00 | 0.00 |
| MNP | MNP_019 | 0.00 | 0.00 | 1.00 | 0.00 |
| MNP | MNP_020 | 0.00 | 0.00 | 1.00 | 0.00 |
| MNP | MNP_021 | 0.00 | 0.00 | 1.00 | 0.00 |
| MNP | MNP_023 | 0.00 | 0.00 | 1.00 | 0.00 |
| MNP | MNP_027 | 0.00 | 0.00 | 1.00 | 0.00 |
| MNP | MNP_028 | 0.00 | 0.00 | 1.00 | 0.00 |
| MNP | MNP_031 | 0.00 | 0.00 | 1.00 | 0.00 |
| MNP | MNP_032 | 0.00 | 0.00 | 1.00 | 0.00 |
| MNP | MNP_033 | 0.00 | 0.00 | 1.00 | 0.00 |
| MNP | MNP_034 | 0.00 | 0.00 | 1.00 | 0.00 |
| MNP | MNP_040 | 0.00 | 0.00 | 1.00 | 0.00 |
| MNP | MNP_041 | 0.00 | 0.00 | 1.00 | 0.00 |
| MNP | MNP_042 | 0.00 | 0.00 | 1.00 | 0.00 |
| MNP | MNP_043 | 0.00 | 0.00 | 1.00 | 0.00 |
| MNP | MNP_055 | 0.00 | 0.00 | 1.00 | 0.00 |
| MNP | MNP_057 | 0.00 | 0.00 | 1.00 | 0.00 |
| MNP | MNP_062 | 0.00 | 0.00 | 1.00 | 0.00 |
| MNP | MNP_063 | 0.00 | 0.00 | 1.00 | 0.00 |
| MNP | MNP_064 | 0.00 | 0.00 | 1.00 | 0.00 |
| MNP | MNP_065 | 0.00 | 0.00 | 1.00 | 0.00 |
| MNP | MNP_095 | 0.00 | 0.00 | 1.00 | 0.00 |
| KIB | Kibwe001 | 0.00 | 0.00 | 1.00 | 0.00 |
| KIB | Kibwe002 | 0.00 | 0.00 | 1.00 | 0.00 |
| KIB | Kibwe003 | 0.00 | 0.00 | 1.00 | 0.00 |
| KIB | Kibwe004 | 0.00 | 0.00 | 1.00 | 0.00 |
| KIB | Kibwe005 | 0.00 | 0.00 | 1.00 | 0.00 |
| KIB | Kibwe006 | 0.00 | 0.00 | 1.00 | 0.00 |
| KIB | Kibwe008 | 0.00 | 0.00 | 1.00 | 0.00 |
| KIB | Kibwe010 | 0.00 | 0.00 | 1.00 | 0.00 |
| KIB | Kibwe011 | 0.00 | 0.00 | 1.00 | 0.00 |
| KIB | Kibwe012 | 0.00 | 0.00 | 1.00 | 0.00 |
| KIB | Kibwe013 | 0.00 | 0.00 | 1.00 | 0.00 |
| KIB | Kibwe016 | 0.00 | 0.00 | 1.00 | 0.00 |
| KIB | Kibwe017 | 0.00 | 0.00 | 1.00 | 0.00 |
| KIB | Kibwe018 | 0.00 | 0.00 | 1.00 | 0.00 |
| KIB | Kibwe019 | 0.00 | 0.00 | 1.00 | 0.00 |
| KIB | Kibwe020 | 0.00 | 0.00 | 1.00 | 0.00 |
| KIB | Kibwe021 | 0.00 | 0.00 | 1.00 | 0.00 |
| KIB | Kibwe022 | 0.00 | 0.00 | 1.00 | 0.00 |
| KIB | Kibwe023 | 0.00 | 0.00 | 1.00 | 0.00 |
| KIB | Kibwe024 | 0.00 | 0.00 | 1.00 | 0.00 |
| KIB | Kibwe025 | 0.00 | 0.00 | 1.00 | 0.00 |
| KIB | Kibwe026 | 0.00 | 0.00 | 1.00 | 0.00 |
| KIB | Kibwe031 | 0.00 | 0.00 | 1.00 | 0.00 |
| KIB | Kibwe032 | 0.00 | 0.00 | 1.00 | 0.00 |
| KIB | Kibwe037 | 0.00 | 0.00 | 1.00 | 0.00 |
| KIB | Kibwe038 | 0.00 | 0.00 | 1.00 | 0.00 |
| KIB | Kibwe039 | 0.00 | 0.00 | 1.00 | 0.00 |
| KIB | Kibwe040 | 0.00 | 0.00 | 1.00 | 0.00 |
| KIB | Kibwe044 | 0.00 | 0.00 | 1.00 | 0.00 |
| TSW | TSWng002 | 0.00 | 0.00 | 1.00 | 0.00 |
| TSW | TSWng013 | 0.00 | 0.00 | 1.00 | 0.00 |
| TSW | TSWng019 | 0.00 | 0.00 | 1.00 | 0.00 |
| TSW | TSWng047 | 0.00 | 0.00 | 1.00 | 0.00 |
| TSW | TSWng048 | 0.00 | 0.00 | 1.00 | 0.00 |
| TSW | TSWng053 | 0.00 | 0.00 | 1.00 | 0.00 |
| TSW | TSWng056 | 0.00 | 0.00 | 1.00 | 0.00 |
| TSW | TSWng065 | 0.00 | 0.00 | 1.00 | 0.00 |
| TSW | TSWng066 | 0.00 | 0.00 | 1.00 | 0.00 |
| TSW | TSWng074 | 0.00 | 0.00 | 1.00 | 0.00 |
| TSW | TSWng076 | 0.00 | 0.00 | 1.00 | 0.00 |
| TSW | TSWng077 | 0.00 | 0.00 | 1.00 | 0.00 |
| TSW | TSWng078 | 0.00 | 0.00 | 1.00 | 0.00 |
| TSW | TSWng080 | 0.00 | 0.00 | 1.00 | 0.00 |
| TSW | TSWng081 | 0.00 | 0.00 | 1.00 | 0.00 |
| TSW | TSWng082 | 0.00 | 0.00 | 1.00 | 0.00 |
| TSW | TSWng084 | 0.00 | 0.00 | 1.00 | 0.00 |
| TSW | TSWng085 | 0.00 | 0.00 | 1.00 | 0.00 |
| TSW | TSWng086 | 0.00 | 0.00 | 1.00 | 0.00 |
| TSW | TSWng087 | 0.00 | 0.00 | 1.00 | 0.00 |
| TSW | TSWng088 | 0.00 | 0.00 | 1.00 | 0.00 |
| TSW | TSWng089 | 0.00 | 0.00 | 1.00 | 0.00 |
| TSW | TSWng091 | 0.00 | 0.00 | 1.00 | 0.00 |
| TSW | TSWng094 | 0.00 | 0.00 | 1.00 | 0.00 |
| TSW | TSWng095 | 0.00 | 0.00 | 1.00 | 0.00 |
| TSW | TSWng096 | 0.00 | 0.00 | 1.00 | 0.00 |
| TSW | TSWng097 | 0.00 | 0.00 | 1.00 | 0.00 |
| TSW | TSWng098 | 0.00 | 0.00 | 1.00 | 0.00 |
| TSW | TSWng099 | 0.00 | 0.00 | 1.00 | 0.00 |
| TSW | TSWng100 | 0.00 | 0.00 | 1.00 | 0.00 |
| KIN | KINny003 | 0.00 | 0.00 | 1.00 | 0.00 |
| KIN | KINny007 | 0.00 | 0.00 | 1.00 | 0.00 |
| KIN | KINny008 | 0.00 | 0.00 | 1.00 | 0.00 |
| KIN | KINny010 | 0.00 | 0.00 | 1.00 | 0.00 |
| KIN | KINny011 | 0.00 | 0.00 | 1.00 | 0.00 |
| KIN | KINny013 | 0.00 | 0.00 | 1.00 | 0.00 |
| KIN | KINny016 | 0.00 | 0.00 | 1.00 | 0.00 |
| KIN | KINny017 | 0.01 | 0.50 | 0.49 | 0.00 |
| KIN | KINny018 | 0.00 | 0.00 | 1.00 | 0.00 |
| KIN | KINny019 | 0.00 | 0.00 | 1.00 | 0.00 |
| KIN | KINny020 | 0.00 | 0.00 | 1.00 | 0.00 |
| KIN | KINny021 | 0.00 | 0.00 | 1.00 | 0.00 |
| KIN | KINny022 | 0.00 | 0.00 | 1.00 | 0.00 |
| KIN | KINny024 | 0.00 | 0.00 | 1.00 | 0.00 |
| KIN | KINny025 | 0.00 | 0.00 | 1.00 | 0.00 |
| KIN | KINny027 | 0.00 | 0.00 | 1.00 | 0.00 |
| KIN | KINny028 | 0.00 | 0.00 | 1.00 | 0.00 |
| KIN | KINny029 | 0.00 | 0.00 | 1.00 | 0.00 |
| KIN | KINny030 | 0.00 | 0.00 | 1.00 | 0.00 |
| KIN | KINny031 | 0.00 | 0.00 | 1.00 | 0.00 |
| KIN | KINny032 | 0.00 | 0.00 | 1.00 | 0.00 |
| KIN | KINny033 | 0.00 | 0.00 | 1.00 | 0.00 |
| KIN | KINny034 | 0.00 | 0.00 | 1.00 | 0.00 |
| KIN | KINny035 | 0.00 | 0.00 | 1.00 | 0.00 |
| KIN | KINny038 | 0.00 | 0.00 | 1.00 | 0.00 |
| KIN | KINny042 | 0.00 | 0.00 | 1.00 | 0.00 |
| KIN | KINny043 | 0.00 | 0.00 | 1.00 | 0.00 |
| KIN | KINny044 | 0.00 | 0.00 | 1.00 | 0.00 |
| KIN | KINny045 | 0.00 | 0.00 | 1.00 | 0.00 |
| KIN | KINny100 | 0.00 | 0.00 | 1.00 | 0.00 |
| SHT | SHti079 | 0.00 | 0.00 | 1.00 | 0.00 |
| SHT | SHti080 | 0.00 | 0.00 | 1.00 | 0.00 |
| SHT | SHti082 | 0.00 | 0.00 | 1.00 | 0.00 |
| SHT | SHti084 | 0.00 | 0.00 | 1.00 | 0.00 |
| SHT | SHti085 | 0.00 | 0.00 | 1.00 | 0.00 |
| SHT | SHti086 | 0.00 | 0.00 | 1.00 | 0.00 |
| SHT | SHti088 | 0.00 | 0.00 | 1.00 | 0.00 |
| SHT | SHti089 | 0.00 | 0.00 | 1.00 | 0.00 |
| SHI | SHm001 | 0.00 | 0.00 | 1.00 | 0.00 |
| SHI | SHm012 | 0.00 | 0.00 | 1.00 | 0.00 |
| SHI | SHm015 | 0.00 | 0.00 | 1.00 | 0.00 |
| SHI | SHm016 | 0.00 | 0.00 | 1.00 | 0.00 |
| SHI | SHm020 | 0.00 | 0.00 | 1.00 | 0.00 |
| SHI | SHm026 | 0.00 | 0.00 | 1.00 | 0.00 |
| SHI | SHm028 | 0.00 | 0.00 | 1.00 | 0.00 |
| SHI | SHm029 | 0.00 | 0.00 | 1.00 | 0.00 |
| SHI | SHm030 | 0.00 | 0.00 | 1.00 | 0.00 |
| SHI | SHpe034 | 0.00 | 0.00 | 1.00 | 0.00 |
| SHI | SHpe035 | 0.00 | 0.00 | 1.00 | 0.00 |
| SHI | SHma039 | 0.00 | 0.00 | 1.00 | 0.00 |
| SHI | SHma044 | 0.00 | 0.00 | 1.00 | 0.00 |
| SHI | SHma045 | 0.00 | 0.00 | 1.00 | 0.00 |
| SHI | SHma046 | 0.00 | 0.00 | 1.00 | 0.00 |
| SHI | SHm006 | 0.00 | 0.00 | 1.00 | 0.00 |
| SHI | SHm007 | 0.00 | 0.00 | 1.00 | 0.00 |
| SHI | SHm017 | 0.00 | 0.00 | 1.00 | 0.00 |
| SHI | SHm018 | 0.00 | 0.00 | 1.00 | 0.00 |
| SHI | SHm031 | 0.00 | 0.00 | 1.00 | 0.00 |
| SHI | SHma038 | 0.00 | 0.00 | 1.00 | 0.00 |
| SHI | SHma040 | 0.00 | 0.00 | 1.00 | 0.00 |
| HND | Hindi001F | 0.00 | 0.00 | 1.00 | 0.00 |
| HND | Hindi001M | 0.00 | 0.00 | 1.00 | 0.00 |
| HND | Hindi002F | 0.00 | 0.00 | 1.00 | 0.00 |
| HND | Hindi002M | 0.00 | 0.00 | 1.00 | 0.00 |
| HND | Hindi003F | 0.00 | 0.00 | 1.00 | 0.00 |
| HND | Hindi003M | 0.00 | 0.00 | 1.00 | 0.00 |
| HND | Hindi004F | 0.00 | 0.00 | 1.00 | 0.00 |
| HND | Hindi004M | 0.00 | 0.00 | 1.00 | 0.00 |
| HND | Hindi005F | 0.00 | 0.00 | 1.00 | 0.00 |
| HND | Hindi005M | 0.00 | 0.00 | 1.00 | 0.00 |
| HND | Hindi006F | 0.00 | 0.00 | 1.00 | 0.00 |
| HND | Hindi006M | 0.00 | 0.00 | 1.00 | 0.00 |
| HND | Hindi007F | 0.00 | 0.00 | 1.00 | 0.00 |
| HND | Hindi007M | 0.00 | 0.00 | 1.00 | 0.00 |
| HND | Hindi008F | 0.00 | 0.00 | 1.00 | 0.00 |
| HND | Hindi008M | 0.00 | 0.00 | 1.00 | 0.00 |
| HND | Hindi009F | 0.00 | 0.00 | 1.00 | 0.00 |
| HND | Hindi009M | 0.00 | 0.00 | 1.00 | 0.00 |
| HND | Hindi010F | 0.00 | 0.00 | 1.00 | 0.00 |
| HND | Hindi010M | 0.00 | 0.00 | 1.00 | 0.00 |
| HND | Hindi011F | 0.00 | 0.00 | 1.00 | 0.00 |
| HND | Hindi011M | 0.00 | 0.00 | 1.00 | 0.00 |
| HND | Hindi012F | 0.00 | 1.00 | 0.00 | 0.00 |
| HND | Hindi012M | 0.00 | 0.00 | 1.00 | 0.00 |
| HND | Hindi013F | 0.00 | 0.00 | 1.00 | 0.00 |
| HND | Hindi013M | 0.00 | 0.00 | 1.00 | 0.00 |
| HND | Hindi014F | 0.00 | 0.00 | 1.00 | 0.00 |
| HND | Hindi014M | 0.00 | 0.00 | 1.00 | 0.00 |
| HND | Hindi015F | 0.00 | 0.00 | 1.00 | 0.00 |
| HND | Hindi015M | 0.00 | 0.00 | 1.00 | 0.00 |
