# Supplementary material for: Phylogeography and population structure of the tsetse fly Glossina pallidipes in Kenya and the Serengeti ecosystem
Source: PLoS Negl Trop Dis. 2020 Feb 24;14(2):e0007855. doi: 10.1371/journal.pntd.0007855 (PMC7058365; doi:10.1371/journal.pntd.0007855)
Supplement: S8 Table — This table shows (a) pairwise genetic differentiation (FST) and (b) geographic distance (in km) among pairs that were not included in Table 3 in the main text. Pairwise FST was computed in Arlequin [44] based on Weir and Cockerham 1984 [65]. Significant values (p > 0.05) are denoted in bold. (DOCX) [file pntd.0007855.s015.docx]

**S8 Table**

| **(a)** |  | **East** | | | | | | | |
| --- | --- | --- | --- | --- | --- | --- | --- | --- | --- |
| **North-west** |  | **NGU** | **MNP** | **KIB** | **TSW** | **KIN** | **SHT** | **SHI** | **HND** |
|  | **KAP** | 0.258 | 0.245 | 0.242 | 0.240 | 0.217 | 0.264 | 0.226 | 0.231 |
|  | **RUM** | 0.258 | 0.291 | 0.288 | 0.304 | 0.266 | 0.312 | 0.263 | 0.282 |
| **Southwest (Serengeti ecosystem)** | **GVR** | 0.128 | 0.177 | 0.190 | 0.188 | 0.167 | 0.143 | 0.169 | 0.184 |
|  | **MRT** | 0.164 | 0.105 | 0.125 | 0.126 | 0.106 | 0.131 | 0.105 | 0.112 |
|  | **FGT** | 0.164 | 0.101 | 0.121 | 0.110 | 0.097 | 0.103 | 0.095 | 0.105 |
|  | **NBS** | 0.145 | 0.157 | 0.168 | 0.166 | 0.147 | 0.115 | 0.148 | 0.159 |
|  | **MRB** | 0.146 | 0.169 | 0.176 | 0.178 | 0.167 | 0.118 | 0.157 | 0.178 |
|  | **GTR** | 0.141 | 0.149 | 0.158 | 0.145 | 0.138 | 0.098 | 0.136 | 0.151 |
|  | **IKR** | 0.130 | 0.154 | 0.172 | 0.160 | 0.148 | 0.118 | 0.149 | 0.164 |
|  | **KLM** | 0.108 | 0.131 | 0.142 | 0.140 | 0.131 | 0.093 | 0.122 | 0.142 |
|  | **MSN** | 0.118 | 0.148 | 0.159 | 0.160 | 0.152 | 0.110 | 0.142 | 0.161 |
|  | **MSS** | 0.127 | 0.156 | 0.169 | 0.166 | 0.151 | 0.112 | 0.142 | 0.162 |
|  | **NGK** | 0.150 | 0.168 | 0.180 | 0.180 | 0.171 | 0.120 | 0.164 | 0.175 |
|  |  |  | | | | | | | |
| **(b)** |  | **East** | | | | | | | |
| **North-west** |  | **NGU** | **MNP** | **KIB** | **TSW** | **KIN** | **SHT** | **SHI** | **HND** |
|  | **KAP** | 347.1 | 423.6 | 535.6 | 603.1 | 740.0 | 788.4 | 809.0 | 787.4 |
|  | **RUM** | 240.6 | 429.5 | 453.8 | 511.9 | 639.7 | 689.7 | 713.2 | 743.1 |
| **Southwest (Serengeti ecosystem)** | **GVR** | 131.1 | 370.9 | 347.5 | 402.5 | 528.5 | 578.6 | 602.4 | 646.9 |
|  | **MRT** | 123.0 | 374.3 | 340.3 | 393.8 | 518.4 | 568.6 | 592.6 | 642.4 |
|  | **FGT** | 109.0 | 361.2 | 326.0 | 380.2 | 505.9 | 556.0 | 579.8 | 627.4 |
|  | **NBS** | 96.0 | 351.5 | 312.8 | 367.4 | 493.6 | 543.7 | 567.4 | 614.3 |
|  | **MRB** | 122.3 | 384.0 | 339.6 | 391.0 | 513.6 | 563.8 | 588.2 | 644.6 |
|  | **GTR** | 198.8 | 481.4 | 405.7 | 445.6 | 553.5 | 603.5 | 629.8 | 719.7 |
|  | **IKR** | 157.0 | 442.4 | 365.5 | 407.7 | 519.4 | 569.6 | 595.5 | 678.6 |
|  | **KLM** | 142.1 | 440.3 | 339.9 | 377.7 | 485.3 | 535.4 | 561.6 | 655.6 |
|  | **MSN** | 210.0 | 509.9 | 396.2 | 426.2 | 522.1 | 571.2 | 598.5 | 713.5 |
|  | **MSS** | 236.7 | 542.1 | 398.2 | 418.2 | 500.7 | 548.5 | 576.4 | 714.3 |
|  | **NGK** | 224.1 | 528.1 | 359.9 | 373.3 | 449.0 | 496.3 | 524.4 | 673.1 |
