# Supplementary material for: Phylogeography and population structure of the tsetse fly Glossina pallidipes in Kenya and the Serengeti ecosystem
Source: PLoS Negl Trop Dis. 2020 Feb 24;14(2):e0007855. doi: 10.1371/journal.pntd.0007855 (PMC7058365; doi:10.1371/journal.pntd.0007855)
Supplement: S9 Table — Home site, sample ID, sex of the migrant, inferred origin of the migrant, and p-value of the test of migrants detected using GENECLASS [70] (a) within the southwest, (b) within the east, (c) between the northwest and southwest, and (d) between the southwest and east. Marginally significant after Benjamini-Hochberg correction for multiple testing (corrected p-value = 0.0308) are marked with *. (DOCX) [file pntd.0007855.s016.docx]

**S9 Table.**

| **(a)** |  |  |  |  |
| --- | --- | --- | --- | --- |
| **Home Site** | **Individual ID** | **Sex** | **Inferred Origin** | ***p-values* (*p*≤0.05)** |
| GVR | MMR148 | Female | MRB | 0.001 |
| GVR | MMR194 | Male | GTR | 0.004 |
| GVR | MMR176 | Male | MSN | 0.001 |
| MRT | MMR075 | Female | FGT | 0.029 |
| MRT | MMR076 | Male | FGT | 0.001 |
| MRT | MMR111 | Male | FGT | 0.030 |
| MRT | MMR118 | Male | FGT | 0.000 |
| MRT | MMR127 | Male | FGT | 0.016 |
| FGT | MMR053 | Male | NBS | 0.034 |
| FGT | MMR011 | Female | MSN | 0.006 |
| NBS | MMR300 | Female | MRB | 0.030 |
| NBS | MMR301 | Female | GTR | 0.034 |
| NBS | MMR299 | Female | NGK | 0.001 |
| MRB | MMR233 | Female | NBS | 0.014 |
| MRB | MMR235 | Female | NBS | 0.021 |
| MRB | MMR265 | Male | GTR | 0.026 |
| MRB | MMR231 | Female | IKR | **0.000*** |
| GTR | GTR003 | Male | NBS | 0.011 |
| GTR | GTR030 | Female | NBS | 0.011 |
| GTR | GTR031 | Female | NBS | 0.044 |
| GTR | GTR008 | Male | IKR | **0.000*** |
| GTR | GTR001 | Male | KLM | 0.050 |
| GTR | GTR034 | Female | MSN | 0.021 |
| IKR | IKR020 | Female | KLM | 0.049 |
| IKR | IKR025 | Female | MSN | 0.045 |
| KLM | SEKF012 | Male | NBS | 0.016 |
| KLM | SEKF029 | Female | MRB | 0.014 |
| KLM | SEKF025 | Female | GTR | 0.003 |
| MSN | MSA027 | Male | GVR | 0.037 |
| MSN | MSA001 | Female | IKR | 0.000 |
| MSN | MSA013 | Female | NGK | 0.037 |
| MSS | MSB014 | Female | GTR | 0.001 |
| MSS | MSB001 | Female | KLM | 0.009 |
| MSS | MSB005 | Female | NGK | 0.019 |
| NGK | NGK030 | Female | GTR | 0.030 |
| NGK | NGK011 | Male | MSS | 0.007 |
| **(b)** |  |  |  |  |
| **Home Site** | **Individual ID** | **Sex** | **Inferred Origin** | ***p-values* (*p*≤0.05)** |
| MNP | MNP_003 | Male | TSW | 0.032 |
| MNP | MNP_021 | Female | TSW | 0.026 |
| MNP | MNP_034 | Male | KIN | 0.043 |
| MNP | MNP_095 | Male | KIN | 0.005 |
| MNP | MNP_002 | Male | SHI | 0.001 |
| KIB | Kibwe006 | Male | MNP | 0.041 |
| KIB | Kibwe032 | Female | KIN | 0.005 |
| KIB | Kibwe017 | Female | SHI | 0.010 |
| KIB | Kibwe026 | Male | SHI | 0.021 |
| KIB | Kibwe013 | Male | HND | 0.002 |
| KIB | Kibwe018 | Female | HND | 0.043 |
| KIB | Kibwe025 | Female | HND | 0.020 |
| TSW | TSWng002 | Female | KIB | 0.039 |
| TSW | TSWng076 | Female | KIN | 0.014 |
| TSW | TSWng091 | Male | KIN | 0.035 |
| TSW | TSWng089 | Female | HND | 0.006 |
| KIN | KINny003 | Female | MNP | 0.032 |
| KIN | KINny029 | Female | MNP | 0.013 |
| KIN | KINny043 | Male | KIB | 0.041 |
| KIN | KINny011 | Male | TSW | 0.042 |
| KIN | KINny028 | Female | TSW | 0.021 |
| KIN | KINny100 | Male | HND | 0.011 |
| SHT | SHti089 | Male | KIB | 0.018 |
| SHT | SHti079 | Male | KIN | 0.038 |
| SHT | SHti082 | Male | KIN | 0.019 |
| SHT | SHti086 | Male | SHI | 0.026 |
| SHI | SHpe035 | Female | TSW | 0.049 |
| SHI | SHm012 | Female | KIN | 0.020 |
| SHI | SHm015 | Female | KIN | 0.044 |
| SHI | SHm040 | Male | SHT | 0.005 |
| SHI | SHm006 | Male | HND | 0.005 |
| SHI | SHm018 | Male | HND | 0.042 |
| HND | Hindi001M | Male | TSW | 0.045 |
| HND | Hindi010F | Female | TSW | 0.015 |
| HND | Hindi011M | Male | TSW | 0.007 |
| HND | Hindi014F | Female | TSW | 0.003 |
| HND | Hindi004M | Male | KIN | 0.005 |
| HND | Hindi005M | Male | KIN | 0.033 |
| **(c)** |  |  |  |  |
| **Home Site** | **Individual ID** | **Sex** | **Inferred Origin** | ***p-values* (*p*≤0.05)** |
| RUM | Ruma_010 | Female | FGT | 0.001 |
| FGT | MMR016 | Female | RUM | 0.004 |
| **(d)** |  |  |  |  |
| **Home Site** | **Individual ID** | **Sex** | **Inferred Origin** | ***p-values* (*p*≤0.05)** |
| NGU | MOK20 | Female | GVR | 0.010 |
| KIN | Kiny_017 | Male | MRB | 0.003 |
| SHT | SHti_086 | Male | MRB | 0.001 |
| SHT | SHti_084 | Male | MSS | 0.000 |
| FGT | MMR015 | Female | NGU | 0.019 |
| GTR | GTR031 | Female | SHT | 0.006 |
| NGK | NGK009 | Male | SHI | 0.002 |
