# Supplementary material for: Phylogeography and population structure of the tsetse fly Glossina pallidipes in Kenya and the Serengeti ecosystem
Source: PLoS Negl Trop Dis. 2020 Feb 24;14(2):e0007855. doi: 10.1371/journal.pntd.0007855 (PMC7058365; doi:10.1371/journal.pntd.0007855)
Supplement: S2 File — Methods of the mitochondrial DNA sequencing done for ABC analysis, and the ABC scenarios used are described. Basic results of the mitochondrial data included, and ABC analysis are reported. (DOCX) [file pntd.0007855.s018.docx]

**Approximate Bayesian Computation (ABC) Methods and Results**

**Methods**

***Mitochondrial DNA sequencing for ABC Analysis***

For approximate Bayesian computation analysis (see below) exploring potential causes of population structure, we sequenced a 439 bp fragment of mitochondrial DNA (mtDNA) from the cytochrome oxidase I gene was PCR-amplified in 24 individuals using C1-J-1751 (5’ – GGA TCA CTG ATA TAG GAT TCC C – 3’) and C1-N-2191 (5’ – CCC GGT AAA ATT AAA ATA TAA ACT TC – 3’). Reactions contained 1-10 ng of template DNA, 2.6 microliters (5X) buffer (GoTaq colorless, Promega, USA), 1.1 μl (10 mM) dNTPs, 0.5 μl (10 mM) primers, 1.1 μl (25 mM) MgCl2, and 0.1 μl (U/μL) GoTaq polymerase, and 6.9 μl of water for a total volume of 13 μl. Thermal cycler (Eppendorf, Germany) parameters were as follows: an initial denaturation step at 95 °C for 5 min, followed by 95 °C for 30 s of denaturation, 40 cycles each for 30 s at 50 °C for annealing, 45 s at 72 °C for extension and a final extension step at 72 °C for 20 min. PCR products were viewed on an agarose gel to confirm size and purity and subsequently cleaned using AMPure XP beads (Beckman Coulter, USA) , as per the manufacturer’s protocol. Sequencing was carried out in both forward and reverse directions at the DNA Analysis Facility on Science Hill at Yale University. Geneious v6.0.6  software [102] was used to edit and align sequences.

***ABC Analysis Scenarios***

Analysis 1 was designed to identify the most likely ancestral lineage: the east in both splits, the east then west in the older and younger splits, the southwest in both splits, or the northwest in both splits (Scenario 1a-4a, respectively; Fig 2). All scenarios had constant N_e_ and unrestrictive time priors bounded by zero.

Analysis 2 was designed to distinguish between the likely timing of splits and N_e_. It compared all possible scenarios of ancestry with constant N_e_ to the same scenario with population reductions in the northwest and southwest (Scenario 1a vs 1b, 2a vs 2b, 3a vs 3b, 4a vs 4b; S3 Table). Population reductions were added to test if this demographic pattern accounted for the mismatch between simulated and observed estimates of Garza-Williamson’s M [87] in Analysis 1 (see below, S2 File). It is well known that there have been massive tsetse control campaigns followed by tsetse resurgence in these regions in the past 100 years [88–90].

For the mtDNA, the mutation model priors were based on published estimates in other closely related insects [101]. For the microsatellites, the mutation model priors were based on the Generalized Stepwise Mutation model [72] matching previous studies in other insects [73] and results from published ABC analysis in *G. pallidipes* [74] and *G. fuscipes* [75]. N_e_ priors were based on previous estimates from microsatellite data [16,26,28,29,74,76,77].

Time priors were conservatively based on the published biogeographic breaks identified in studies of animals with similar distributions (e.g. mosquitoes [25]; termites [78], African spitting cobras [79]) and through meta-analysis [80,81]. Time priors were converted from “years ago” to “generations ago” using the average generation time of *G. pallidipes* of 5 per year (75 days; [74,82–84]. The younger divergence between the northwest and southwest clusters (Fig 2: t1) was allowed to vary from 0-8.5 million years ago (estimated time of uplifting of the Kenyan highlands north of the break between the Sudanian and Zambesian biogeographic regions [80,81][85,86]). The older divergence between the west and east (Fig 2: t2) was constrained to values greater than t1, but was otherwise allowed to vary from 0-16 million years ago (estimated time of the opening of the Great Rift Valley “Ethiopian Rift” [25,78,79][85,86]).

Accuracy of the mtDNA ABC analysis was assessed using 21 summary statistics: number of haplotypes, number of segregating sites, mean of pairwise differences, variance of pairwise differences, Tajima’s D, number of pairwise shared haplotypes, and pairwise F_ST_ distances [53]. Accuracy of the microsatellite ABC analysis was assessed using 18 summary statistics: Number of alleles, mean heterozygosity, and the M-index [41,87] for each lineage, and the mean number of alleles, mean genetic diversity, and mean pairwise F_ST_ distances [53]. PCA was then performed on these 18 summary statistics to estimate the posterior probability of each scenario relative to observed data with the weighted logistic regression method described by [91].

**Results**

***Mitochondrial DNA sequencing Results***

The 24 mitochondrial sequences generated for the ABC analysis fell into 10 haplotypes, and the most common Haplotype 1 was found in all groups of samples selected to represent the east, northwest, and southwest. All other haplotypes were unique to one of the genetic clusters: Haplotype 2 in the northwest, Haplotype 3 in the southwest, and Haplotypes 4-10 in the east (S5 Fig).

***ABC Analysis Results***

In Analysis 1, there was no clear winning scenario in either the mtDNA or microsatellite datasets, with low posterior probabilities and high posterior predictive error (S3 Table). There was also an under-estimation of F_ST_ in the mtDNA analysis (S3 File) and an over-estimation of mean Garza-Williamson’s M in the microsatellite analysis (S4 File) in the northwest and southwest, indicating more population reductions in the northwest and southwest than what was modeled. Because there was no clear winning scenario in Analysis 1, we proceeded with Analysis 2 designed to distinguish the most likely demographic history and timing of population splits using all four patterns of ancestry possible (Scenarios 1a vs 1b, 2a vs 2b, 3a vs 3b, and 4a vs 4b; S1 Fig).

Analysis 2 indicated that Scenarios 1b, 2b, 3b, and 4b allowing Ne to vary (S1 Fig) had much higher posterior probabilities than the alternative Scenarios 1a, 2a, 3a, and 4a, respectively (S3 Table). These results suggest that there were indeed population reductions in the northwest and southwest within the last century. Posterior predictive error in Analysis 2 averaged 0.213 in the mtDNA analysis, and 0.109 in the microsatellite analysis (S3 Table. Most scenario comparisons indicated that scenarios allowing Ne to vary (Scenarios 1a, 2a, 3a, and 4a) were more realistic than scenarios with constant Ne (Scenarios 1b, 2b, 3b, and 4b).

Parameter estimates from the mtDNA analysis (S7 Fig) suggested that divergence between the northwest and southwest was occurred within the last several years (in other words was consistent with being caused by IBD rather than a biogeographic break). Divergence between the west and east was predicted to have occurred between 1 and 13 mya, and the mtDNA mutation rate was estimated within the range of 2.65E-07 to 1.00E-05. However, parameter estimates may not be reliable because of the high posterior predictive error.

Parameter estimates from the microsatellite analysis generally agreed with the mtDNA analysis, and were consistent with an average microsatellite mutation rate in the range of 1.35E-04 to 5.66E-04, and a geometric distribution of repeat length mutation step size of about 0.3. However, we do not attempt to interpret time estimates from the microsatellite analysis because these were likely unreliable because of the high posterior predictive error, and because microsatellites generally have fast mutation rates that make them inappropriate to estimate timing of splits on the order of millions of years ago (S7 Fig).
